# Differential transcriptome response of blood brain barrier spheroids to neuroinvasive *Neisseria* and *Borrelia*

**DOI:** 10.3389/fcimb.2023.1326578

**Published:** 2023-12-19

**Authors:** Amod Kulkarni, Jana Jozefiaková, Katarína Bhide, Evelína Mochnaćová, Mangesh Bhide

**Affiliations:** ^1^Laboratory of Biomedical Microbiology and Immunology, The University of Veterinary Medicine and Pharmacy, Kosice, Slovakia; ^2^Institute of Neuroimmunology of Slovak Academy of Sciences, Bratislava, Slovakia

**Keywords:** blood-brain barrier, BBB-spheroids, *Neisseria meningitidis*, *Borrelia bavariensis*, transcriptome analysis, human BMECs, pericytes, astrocytes

## Abstract

**Background:**

The blood-brain barrier (BBB), a highly regulated interface between the blood and the brain, prevents blood-borne substances and pathogens from entering the CNS. Nevertheless, pathogens like *Neisseria meningitidis* and *Borrelia bavariensis* can breach the BBB and infect the brain parenchyma. The self-assembling BBB-spheroids can simulate the cross talk occurring between the cells of the barrier and neuroinvasive pathogens.

**Methods:**

BBB spheroids were generated by co-culturing human brain microvascular endothelial cells (hBMECs), pericytes and astrocytes. The BBB attributes of spheroids were confirmed by mapping the localization of cells, observing permeability of angiopep2 and non-permeability of dextran. Fluorescent *Neisseria*, *Borrelia* or *E. coli* (non-neuroinvasive) were incubated with spheroids to observe the adherence, invasion and spheroid integrity. Transcriptome analysis with NGS was employed to investigate the response of BBB cells to infections.

**Results:**

hBMECs were localized throughout the spheroids, whereas pericytes and astrocytes were concentrated around the core. Within 1 hr of exposure, *Neisseria* and *Borrelia* adhered to spheroids, and their microcolonization increased from 5 to 24 hrs. Integrity of spheroids was compromised by both *Neisseria* and *Borrelia*, but not by *E. coli* infection. Transcriptome analysis revealed a significant change in the expression of 781 genes (467 up and 314 down regulated) in spheroids infected with *Neisseria*, while *Borrelia* altered the expression of 621 genes (225 up and 396 down regulated). The differentially expressed genes could be clustered into various biological pathways like cell adhesion, extracellular matrix related, metallothionines, members of TGF beta, WNT signaling, and immune response. Among the differentially expressed genes, 455 (48%) genes were inversely expressed during *Neisseria* and *Borrelia* infection.

**Conclusion:**

The self-assembling spheroids were used to perceive the BBB response to neuroinvasive pathogens - *Neisseria* and *Borrelia*. Compromised integrity of spheroids during *Neisseria* and *Borrelia* infection as opposed to its intactness and non-adherence of *E. coli* (non-neuroinvasive) denotes the pathogen dependent fate of BBB. Genes categorized into various biological functions indicated weakened barrier properties of BBB and heightened innate immune response. Inverse expression of 48% genes commonly identified during *Neisseria* and *Borrelia* infection exemplifies unique response of BBB to varying neuropathogens.

## 1 Introduction

The blood-brain barrier (BBB) regulating the influx and efflux of ions, oxygen, nutrients, metabolites and cells between the blood and brain parenchyma protects the central nervous system from toxic and pathogenic insults. BBB encompasses a monolayer of specialized endothelial cells lining the cerebral microvessels. Brain microvascular endothelial cells (BMCEs) are interconnected by a paracellular cleft sealed by adherent and tight junctional proteins that aid in producing transendothelial resistance restricting paracellular transfer of molecules into the brain parenchyma. Moreover, high mitochondrial content, limited transcytotic vesicles and absence of fenestrae in the BMCEs synergizes the restriction on blood compounds to gain CNS entry (Abbott et al., 2010). The barrier property of BBB is further supported by pericytes and astrocytes which are in close anatomical proximity with BMECs. Pericytes are the perivascular cells enclosing the BMECs at the basement membrane and share the same extracellular matrix - basal lamina (Abbott et al., 2010; Coureuil et al., 2017). Pericytes are known to regulate the barrier function of BBB by maintaining junctional architecture of endothelium and restrict over production of pinocytotic vesicle in BMECs (Armulik et al., 2010). Astrocytes connect with BMECs through their endfeet projections which surround the capillaries and venules within the brain parenchyma, and perform sieving function on fluid and solute flux (Iliff et al., 2012). The extracellular matrix shared by the BMCEs and astrocyte endfeet projection - basal lamina 2 differs in composition from basal lamina 1 (Abbott et al., 2010). Moreover, the perivascular space between endothelial cells and astrocyte endfeet is filled with the interstitial fluid which is known to possess lymphatic function (Iliff et al., 2012; Coureuil et al., 2017). These three cell types (BMECs, pericytes and astrocytes) along with the smooth muscle cells and neurons are interlinked tightly to the extracellular matrix of basal lamina forming the neurovascular unit (NVU) (Jessen et al., 2015).

The complex architectural characteristics of the neurovascular unit aids in limiting the traffic of both pathogens and host immune cells such as granulocytes and lymphocytes to enter the brain paranchyma. Nevertheless, several pathogens can invade the BBB and cause chronic neurological manifestations (e.g. *Borrelia garinii, Borrelia bavariensis*), while some cause acute meningitis (like *Neisseria meningitidis, Streptococcus Sp., Listeria monocytogenes*) as reviewed elsewhere (Bencurova et al., 2011; Pulzova et al., 2011). Transmigration of *N. meningitidis* and *B. bavariensis* through human BMECs (hBMECs) occurs through transcellular receptor-mediated endocytosis or by paracellular crossing (Comstock and Thomas, 1989; Grab et al., 2005; Coureuil et al., 2010; Divan et al., 2018). Platelet activating factor receptor (PAFR), laminin receptor, poly-Ig receptor (pIgR), PCAM-1, CD147, β2‐adrenergic receptor and plasminogen activator receptor are some of the well-known receptors on BMCEs targeted for transcellular receptor-mediated endocytosis (Comstock and Thomas, 1989; Grab et al., 2005; Orihuela et al., 2009; Al-Obaidi and Desa, 2018). On the other hand, disruption of inter-cellular tight junctions by enzymes belonging to fibrinolytic system and matrix metalloproteinases (e.g. MMP1 and MMP8) during *N. meningitidis* and *B. burgdorferi* infection in hBMECs denotes paracellular passage of bacteria (Schubert-Unkmeir et al., 2010). Permeability of hBMCEs during meningococcal infection has been further confirmed through high throughput sequencing methods, in which transcripts of the molecules involved in cell adhesion, endocytosis, cytoskeletal and extracellular matrix re-organization, cytokine production, pattern recognition receptors dependent signaling (e.g. TLR-2), apoptosis and metabolism were prominently evoked (Nassif et al., 2002; Schubert-Unkmeir et al., 2007; Coureuil et al., 2012; Coureuil et al., 2017; Al-Obaidi and Desa, 2018; Káňová et al., 2019). Interaction of *Borrelia* with BMECs, microglia, astrocytes, brain tissues of non-human primates and mice has also shown to evoke MMPs, transcription factors and inflammatory and chemokine pathways (Ramesh et al., 2003; Ramesh et al., 2008; Brissette et al., 2013; Carey and Ho, 2017; Casselli et al., 2017). Overexpression of chemokines in astrocytes was suggested to promote infiltration of inflammatory cells causing neuronal damage during neuroborreliosis (Ramesh et al., 2003; Ramesh et al., 2008). Most of the aforementioned studies describing the molecular events in BBB invasion have used single cell types (e.g. BMCEs or glial cells) and the conclusions were drawn towards the BBB permeability. Although, the transwell systems with the monolayer of endothelial cells or choroid plexus epithelial cells are efficient *in vitro* models to study neuroinvasive bacterial pathogenesis (Grab et al., 2005; Grab et al., 2009; Martins Gomes et al., 2019; Thompson et al., 2020). It is apparent that non-endothelial cells of the NVU are obligatory to generate the complete BBB phenotype.

The co-culture of BMCEs, pericytes, and astrocytes, which forms spontaneous 3D spheroids and simulates the BBB, has recently been standardized (Bergmann et al., 2018). The BBB spheroids bear several distinct features of the barrier like the presence of appropriate junctional proteins and efflux/influx pumps, direct contact between different NVU cell, reduced to nil paracellular permeability and evidence for receptor-mediated transcytosis (Cho et al., 2017; Gastfriend et al., 2018; Kumarasamy and Sosnik, 2021). Thus, in the present study, BBB spheroids produced by co cultivation of human BMECs (hBMECs), human brain vascular pericytes (pericytes) and human astrocytes (astrocytes) were used to observe the infection of *N. meningitidis* (*Neisseria*) and *B. bavariensis* (*Borrelia*) using time lapse microscopy. Further, differential transcriptome analysis of spheroids exposed to *Neisseria* or *Borrelia* was performed through illumina RNAseq technology to elucidate the BBB’s response to invading pathogens.

## 2 Materials and methods

### 2.1 Bacterial culture

*N. meningitidis* (isolate M1/03, MC58 homologue, Serogroup B) was cultured in brain heart infusion broth (BHI; Jena Bioscience, Germany) containing 10 mM MgCl_2_, (Sigma, Slovakia) at 37 °C in 5% CO_2_.

To stain *Neisseria*, culture (OD_600_ 0.6) was centrifuged (3420 x g for 10 min), pellet was redispersed in 2 mL BHI broth with 1x SYBR green I dye (Thermo Fisher Scientific, USA) and incubated for 2 h (37°C, 5% CO_2_). Bacteria were washed with 1x PBS and redispersed into 1 mL of EBM-2 basal medium (Lonza, Switzerland). *Neisseria* were enumerated using the flow cytometer (flow rate: slow, 1 min, gate at FL1 533/30nm versus SSC-A threshold of 10,000 on BD Accuri C6, USA).

Neuroinvasive strain of the *B. bavariensis* sp. *nov.* strain SKT-7.1 constitutively expressing eGFP (Pulzova et al., 2013) was grown in 40 mL of BSK-II medium till its logarithmic phase, centrifuged at 3420 x *g* for 10 min, the pellet was washed with 1x PBS (three times) and resuspended in 4 mL of EBM-2 medium. *Borrelia* were counted using the flow cytometer (flow rate slow, 1 min, core size 10 μm, gate SSC-H threshold 5000 versus FL1 510/15nm threshold 3000).

*E. coli* strain SG13009 a non-biohazardous laboratory strain [sold by Qiagen, Germany for protein overexpression, (Gottesman et al., 1981)], was modified to express eGFP (Comor et al., 2017). *E. coli* were grown in 10 mL of LB medium (OD_600_ 0.6) with 50 μg/mL carbenicillin, centrifuged (3420 x *g* for 10 min) and pellet was resuspended in 2 mL of EBM-2 medium. *E. coli* were counted on the flow cytometer using the identical parameters used for *Borrelia.*

### 2.2 Cell culture conditions

Human brain microvascular endothelial cells (hBMCE/D3, Merck, Czech Republic) were cultured in DMEM-F12 medium as described previously (Hruškovicová et al., 2022). Human brain vascular pericytes (pericytes, ScienCell, USA) were cultured on Poly-L-Lysine (2 µg/cm^2^, Sigma Aldrich) coated flask in pericyte medium supplemented with 2% FBS, 1x pericyte growth supplements and penicillin-streptomycin (all components from ScienCell). Human astrocytes (ScienCell) were cultured on Poly-L-Lysine (2 µg/cm^2^) coated flask in DMEM high glucose GlutaMax (Thermo Fisher Scientific) supplemented with 10% FBS, 1x N2-supplement (Thermo Fisher Scientific) and human epidermal growth factor (20 ng/mL, Merck). All cell types were maintained between 3^rd^- 6^th^ passage and were cultured at 37 °C, 5% CO_2_ until 80-85% confluency.

### 2.3 *In vitro* culture of BBB spheroids

Self-assembling multicellular spheroids were produced as described previously (Bergmann et al., 2018). In brief, 50 µL of molten 1% agarose in PBS (w/v) was dispensed in 96-well plates. After solidification, 200 µL of BBB-working medium (2% human serum (v/v) in EGM-2 Endothelial Cell Growth Medium-2 BulletKit, Lonza, Switzerland) was added in each well. VEGF was omitted from the BBB-working medium to avoid tight junction disruption (Bergmann et al., 2018). hBMECs, pericytes, and astrocytes cultured in T-75 flask (85% confluency) were dislodged (trypsin-EDTA, Biowest, France) and washed once with EBM-2 medium. All cell types were mixed in a ratio 1:1:1 and seeded on agarose coated well (4.5 x 10^3^ cells/well). Cells were incubated at 37 °C in 5% CO_2_ until spheroid formation.

### 2.4 Localization of hBMECs, astrocytes and pericytes in BBB spheroids

CellTracker deep red (1µM, 630nm_ex_/650nm_em_) was used to stain hBMECs, while CellTracker green BODIPY (10µM, 522nm_ex_/529nm_em_), and CellTracker blue CMHC (10µM, 353nm_ex_/466nm_em_) were used to stain pericytes and astrocytes, respectively, according the manufacturers’ instructions (all dyes are from Thermo Fisher Scientific). Stained cells were seeded on agarose coated well in 1:1:1 ratio (4.5 x 10^3^/well) and incubated until spheroid formation. Few spheroids were frozen in OCT mounting medium (VWR) and cryosections of 10 microns were cut on LEICA biosystem. The cryosections were mounted on poly-l-lysin coated microscope slides (Thermo Scientific), fixed with cold acetone for 10 min and mounted with Fluoroshield (Sigma Aldrich) prior to their photo-documentation on Cytation 7 (BioTek, USA) or LSM700 confocal microscope (Zeiss, Germany).

### 2.5 Integrity of self-assembled spheroids governed by tight junctions

Integrity of self-assembled spheroids was examined by comparing the permeability of angiopep-2 and dextran. Ten spheroids were transferred to ultra-low attachment 96 well plate containing 200 µL of BBB-working medium with angiopep-2 5-TAMRA conjugate (10 µg/mL, Lumiprobe, Germany) or 70 kDa dextran-FITC (10 µg/mL, Thermo Fisher Scientific). Spheroids were incubated for 24 h at 37 °C in CO_2_ incubator with constant rotation (5 rpm).

To confirm the integrity of spheroid governed by tight junctions, three spheroids were incubated with different concentrations of VEGF-A (25, 50 and 100 ng/mL) and dextran-FITC (10 µg/mL) for 24 h at 37 °C in CO_2_ incubator. Spheroids were then washed with 200 µL of 1x PBS (7 rpm, 10 min; 5 times) and fixed in 4% formalin at room temperature for 1 h. Spheroids were re-washed with PBS, laid on coverslip, air dried and placed within the cavity of glass depression slides. Photo documentation was performed on Cytation 7 using 542nm_ex_/568nm_em_ for angiopep-2-5-TAMRA and 495nm_ex_/518nm_em_ for dextran-FITC.

### 2.6 Adhesion of *Neisseria* and *Borrelia* on spheroids

Spheroids in 200 µL of BBB-working medium devoid of antibiotics were challenged with either 6x10^3^ SYBR green I stained *Neisseria* (MOI = 1:4; number of hBMECs used to make spheroid: *Neisseria*) or 1.5x10^4^ constitutively expressing eGFP *Borrelia* (MOI = 1:10) or non-neuroinvasive *E. coli* expressing eGFP (MOI = 1:4). Time lapse microscopy was performed to visualize the bacterial adhesion using Cytation 7. Images of infected spheroids were captured at 1, 3, 5 and 24 h post exposure using 495nm_ex_ and 518nm_em_ to visualize the green fluorescence of bacteria.

### 2.7 Integrity of spheroids after exposure to *Neisseria* or *Borrelia*

Spheroids were exposed to either *Neisseria*, *Borrelia*, or *E. coli* under the same conditions as described above. Dextran-tetramethylrhodaimine (70 kDa, 10 µg/mL) was immediately added to all wells and incubated in a CO_2_ incubator at 37°C with rotation (5 rpm). After 3 h, the spheroids were washed with PBS and fixed with the 4% formalin as described above. The passage of dextran-tetramethylrhodaimine in spheroids was analyzed using Cytation 7 at 555 nm_ex_ and 580 nm_em_.

### 2.8 Cell response of spheroids to *Neisseria* or *Borrelia* infection

As previously described, 144 spheroids were generated and transferred to ultra-low attachment plates containing 200 µl of BBB-working medium free of hydrocortisone, GA-100, and ascorbic acid. Simultaneously, *Neisseria* culture (OD_600_ 0.6) and *Borrelia* culture in its logarithmic phase was centrifuged (3420 x *g* for 10 min) and the pellet was resuspended in 2 mL of EBM-2 basal medium. Bacteria were enumerated on flowcytometer as described before. Spheroids (N= 36) were challenged with either *Neisseria* (6 x 10^4^, MOI 1:4) or *Borrelia* (1.5 x 10^5^, MOI 1:10). Spheroids without infection served as control group (**Supplementary Figure 1A****).** After 3 h, 12 spheroids per treatment were pooled in a tube and three pools (replicates) for each treatment were designated. Pooled spheroids were rinsed with PBS (in nuclease free water) and the total RNA was extracted using RNeasy Mini Kit (Qiagen), which includes on-column DNase digestion. Concentration of RNA was measured on NanoDrop and the integrity was assessed on 1% agarose gel electrophoresis. RNA (500 ng) was reverse transcribed using oligodT primer included in QuantSeq FWD 3’mRNA Library Prep Kit (Lexogen, Austria). Half the recommended volume of OligodT primer (2.5 µL) was used to minimize the off-target products. RNA removal solution (RS buffer, Lexogen) was used on cDNA to eliminate any remaining RNA. Second strand was synthesized using the UMI Second Strand Synthesis Mix (USS, Lexogen) containing 6 nucleotides long Unique Molecular Identifiers (UMIs). UMI allows detection and removal of PCR duplicates. The double stranded cDNA libraries were amplified in 17-19 PCR cycles using i5 Unique Dual Indexing Add-on Kit (Lexogen). The quality and quantity of libraries was determined on Fragment Analyzer using DNF-474 HS NGS Fragment Kit (Agilent Technologies) and QuantiFluor dsDNA System (Promega), respectively. Illumina NextSeq 500 was employed to sequence the libraries having a read lengths of single-end 75 bp producing about 10 million reads per library (**Supplementary Table 1**).

### 2.9 Data analysis

High-throughput RNA-Seq data obtained from sequenced libraries in Bcl file format was converted to Fastq format using bcl2fastq v. 2.20.0.422 (Illumina software for basecalling). The 6-nucelotide long UMIs were extracted and the deduplication of aligned reads was performed by UMI-tools v. 1.1.1. as described elsewhere (Smith et al., 2017). Trimming of UMI and the quality assessment of raw single-end fastq reads was performed using seqtk 1.3 (r106) (https://github.com/lh3/seqtk/releases/tag/v1.3) and FastQC v0.11.9 (http://www.bioinformatics.babraham.ac.uk/projects/fastqc/) respectively. Trimmomatic v0.36 (https://github.com/usadellab/Trimmomatic) was used to trim the adapter sequence and raw fastq reads. The resulting trimmed RNA-Seq reads were mapped to human genome (hg38) and Ensembl GRCh38 v.94 annotation using STAR v2.7.3a (https://github.com/alexdobin/STAR). Quality control on mapped reads (number and percentage of uniquely and multi-mapped reads, rRNA contamination, mapped regions, read coverage distribution, strand specificity, gene biotypes and PCR duplication) was performed using tools: RSeQC v2.6.2 (http://rseqc.sourceforge.net/), Picard v2.18.27 (https://github.com/broadinstitute/picard), Qualimap v.2.2.2 (http://qualimap.conesalab.org/) and BioBloom tools v 2.3.4-6-g433f (https://github.com/bcgsc/biobloom).

To carryout gene expression analysis, Bioconductor R-package - DESeq2 v1.20.0 (Love et al., 2014) was used where in, featureCounts tool v1.6.3 (Liao et al., 2013) was used to enumerate the genes. A minimum threshold of adjusted p-value < 0.05 and log_2_fold-change (log_2_FC) = ± 1 was set to considered the gene as differentially expressed (DEG). RNA-seq data was deposited into publicly available EBI Arrayexpress repository (Accession no E-MTAB-13401).

### 2.10 Bioinformatics analysis of differentially expressed genes

Log_2_FC values of DEGs were used to create a violin plot (Graphpad Prism v9.3). DEGs were analyzed on Reactome (https://reactome.org/) to segregate them in various biological processes (cutoff p-value < 0.05). Genes unclassified in Reactome, were examined in DAVID Bioinformatics Resources v 6.8 (https://david.ncifcrf.gov/home.jsp) to determine additional clusters (threshold for entry p value set as < 0.05). A Venn diagram was used to determine the relationship between the DEGs found in both infections (Venny 2.1 https://bioinfogp.cnb.csic.es/tools/venny/) and heat maps to visualize the clustered DEGs (GraphPad).

### 2.11 Validation of DEGs by quantitative real-time polymerase chain reaction

Reverse transcription of RNA (1 µg) into cDNA was performed using random hexamer primers as described previously (Bhide et al., 2022). mRNA levels of DEGs were corroborated with qPCR using 6 ng of cDNA, 10 pmol of gene specific primers (**Supplementary Table 2**, primers designed with Geneious Pro software) and Sybr green chemistry on StepOnePlus Real-Time PCR System (Bhide et al., 2022). The average of threshold cycle (Ct) values of two reference genes - β2-microtubulin and glyceraldehyde 3-phosphate dehydrogenase was used for normalization. The relative gene expression (2^-ΔΔCt^) and log_2_fold change (Log_2_FC) was determined as described before (Bhide et al., 2022). Pearson correlation coefficient (r) was calculated between the Log_2_FC values of RNA-seq and qRT-PCR using Graphpad.

## 3 Results

### 3.1 Self-assembly of spheroids and their integrity

**Figure 1:**
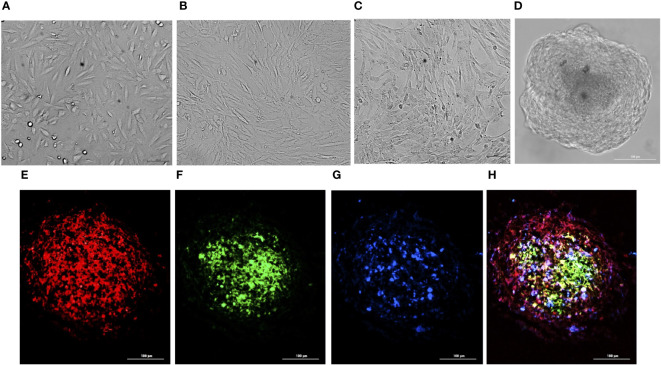
Preparation of BBB spheroids. Bright-field images of hBMECs **(A)**, pericytes **(B)**, and astrocytes **(C)** self-assembled into BBB spheroids **(D)**. Fluorescence images of cryosection (10 µm) of spheroid generated by co-culturing hBMECs pre-stained with CellTracker Deep Red, captured at 630nm_ex_/650nm_em_ **(E)**, pericytes pre stained with CellTracker Green BODIPY, captured at 522nm_ex_/529nm_em_ **(F)**, astrocytes pre-stained with CellTracker Blue CMHC captured 353nm_ex_/466nm_em_ **(G)**, and a merged image **(H)** generated by combining **(E–G)**.

**Figure 2:**
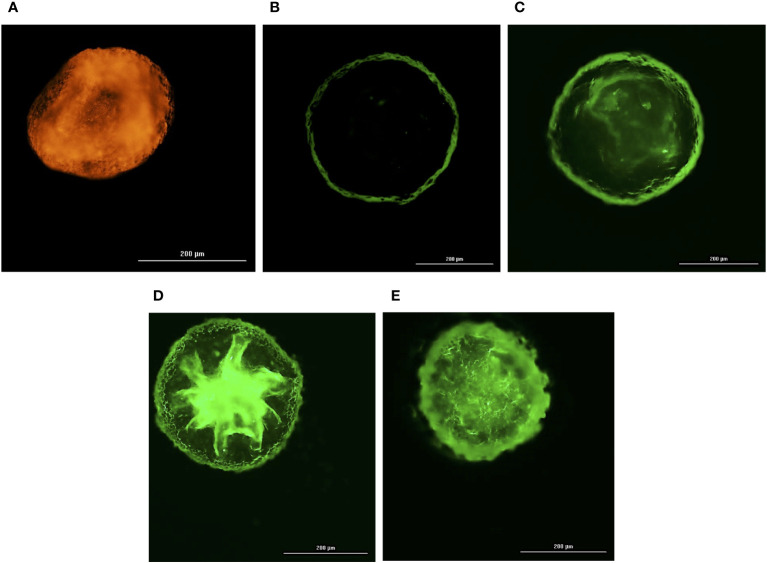
Integrity of self-assembled spheroids. Fluorescence images of a 4% formalin fixed BBB spheroids pre-incubated with 10 µg/mL of angiopep-2 5-TAMRA conjugate **(A)**, 10 µg/mL of 70kDa dextran – FITC **(B)**, 25 ng/mL of VEGF-A and 10 µg/mL of 70kDa dextran – FITC **(C)**, 50 ng/mL of VEGF-A and 10 µg/mL of 70kDa dextran – FITC **(D)**, 100 ng/mL of VEGF-A and 10 µg/mL of 70kDa dextran – FITC **(E)** for 24h. Orange fluorescence of angiopep-2 5-TAMRA conjugate observed at 542nm_ex_/568nm_em_ **(A)** denotes permeation of angiopep-2, whereas green fluorescence of FITC observed on the periphery of spheroid captured at 495nm_ex_/518nm_em_ denotes non traversal of 70kDa dextran – FITC **(B)**. However, simultaneous incubation of VEGF-A (25-100 ng/mL) and10 µg/mL of 70kDa dextran – FITC **(C–E)** shows corresponding increase in green fluorescence of FITC depicting VEGF-A dependent permeability of 70kDa dextran.

All the three co-cultured cell types, hBMECs, pericytes and astrocytes, aggregated within 48 h to form a single spheroid (**Figures 1A–D**). Microscopy of spheroids cultured with cell-tracker-stained cells revealed that hBMECs are present throughout the spheroid, whereas pericytes and astrocytes are concentrated around the core (**Figures 1E–H**; **Supplementary Images 1** and **2**). The barrier function of the endothelial cell layer was confirmed by incubating spheroids with dextran-FITC or angiopep-2 5-TAMRA conjugate. Dextran was only found on the periphery, whereas angiopep-2 was observed throughout the spheroid (**Figures 2A, B**). Incubation of spheroids with VEGF-A increased dextran influx in a concentration-dependent manner (**Figures 2C-E**). As VEGF is known to disrupt intercellular junctions and increase paracellular permeability (Esser et al., 1998; Antonetti et al., 1999) the findings show that intercellular adherent, tight, and gap junctions were formed properly and spheroids were suitable to use in further experiments.

### 3.2 Adhesion of bacteria on spheroids and its effect on integrity

**Figure 3:**
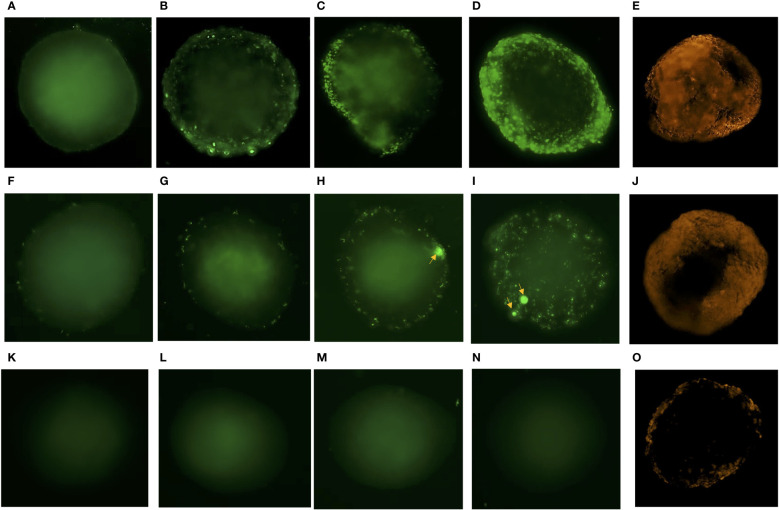
Time lapse microscopy of spheroids exposed to *Neisseria*, *Borrelia*, *E. coli* and evaluation of their integrity. Representative images of BBB spheroids exposed to SYBR green I stained *Neisseria* captured at 1h **(A)**, 3h **(B)**, 5h **(C)** and 24h **(D)** post infection at 495nm_ex_/518nm_em_ is depicted. **(E)** Infusion of 70 kDa dextran – tetramethylrhodaimine in spheroid exposed to *Neisseria* for 3h observed as orange fluorescence captured at 555 nm_ex_/580 nm_em_ depicts compromised integrity of BBB spheroids during infection. Similarly, representative images of spheroids exposed to constitutively expressing eGFP *Borrelia* captured at 1h **(F)**, 3h **(G)**, 5h **(H)** and 24h **(I)** post infection and **(J)** infusion of dextran – tetramethylrhodaimine in spheroids at 3h is depicted. Representative images of spheroids exposed to non-neuroinvasive *E. coli* expressing eGFP captured at 1h **(K)**, 3h **(L)**, 5h **(M)** and 24h **(N)** is depicted. Meager adhesion of 70 kDa dextran – tetramethylrhodaimine on the periphery of spheroids **(O)** exposed to *E. coli* for 3h shows optimum integrity of spheroids. Increase in adhesion **(B)**, multiplication **(C, D)** of *Neisseria* and aggregation of *Borrelia* potentially forming biofilm (**H, I**; indicated by arrows), with the passage of time (1-24h) is evident as opposed to no adhesion of *E. coli* **(K–N)**.

In time-lapse microscopy of spheroids exposed to fluorescent *Neisseria* and *Borrelia*, adherence was observed within the 1^st^ hour of incubation, (**Figures 3A, F**). A marginal increase in their adhesion was observed by 3 hours of incubation (**Figures 3B, G**). By the 5^th^ hour of exposure, there was micro-colonization of *Neisseria* and aggregation of *Borrelia* possibly forming biofilm (**Figures 3C, H**) (Sapi et al., 2012). By the 24^th^ hour, there was heavy colonization of *Neisseria* and increased aggregation of *Borrelia* throughout the spheroids (**Figures 3D, I**). *E. coli*, on the other hand, did not adhere the spheroid even after 24 h of incubation (**Figures 3K–N**). Infection of *Borrelia* and *Neisseria* compromised the integrity and barrier properties of spheroids as the influx of the dextran-tetramethylrhodaimine was clearly observed by 3 hours post incubation (**Figures 3E, J**). On the other hand, spheroids exposed to *E. coli*, showed the meager presence of dextran at the periphery (**Figure 3O**).

### 3.3 RNAseq data analysis, validation and bioinformatic analysis

**Figure 4:**
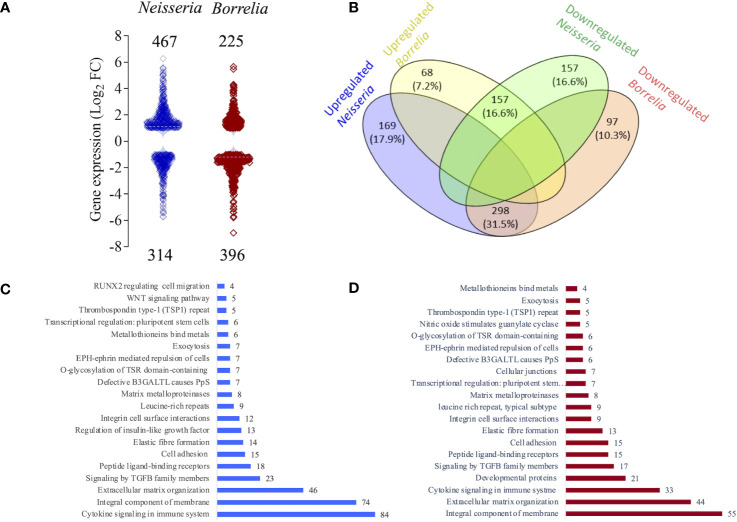
Classification of differentially expressed genes (DEGs). **(A)** Violin plot depicting the distribution of significantly up (Log_2_FC >1) or down regulated genes (Log_2_FC <1) in spheroids infected with either *Neisseria* (blue) or *Borrelia* (maroon) identified by RNAseq. Log_2_FC of each gene is represent by a square and total number of DEGs are mentioned. **(B)** Venn diagram showing the relationship between the DEGs identified during *Neisseria* or *Borrelia* infection. Blue and green ellipse represent number and percentage of up and down regulated DEGs during *Neisseria* infection respectively. Likewise yellow and orange ellipse represents number and percentage of up and down regulated DEGs during *Borrelia* infection respectively. Number and percentage of commonly expressed DEGs are mentioned in the intersection of ellipses. Bar graphs showing 20 salient clusters and number of DEGs classified within them during *Neisseria* **(C)** or *Borrelia* **(D)** infection.

The response of the BBB spheroid undergoing *Neisseria* or *Borrelia* infection was mapped using RNAseq. RNA extracted from the infected and non-infected spheroids had suitable concentration and quality necessary for the synthesis of cDNA libraries (**Supplementary Figures 1B, C**). The fragment size in all 12 cDNA libraries was about 220 -250 bp (**Supplementary Figures 2, 3**). Raw reads produced from NGS ranged from 9.3 to 13.0 million. Approximately 73-79% of processed reads were uniquely mapped to the human genome. **Supplementary Table 1** contains additional information about the reads and their mapping to the human genome. A total of 30,145 genes were mapped from sequenced libraries with more than one normalized read (**Supplementary Data Sheet 1**). The expression of 781 genes was significantly altered (DEGs) in spheroids challenged with *Neisseria* (467 genes with Log_2_FC ranged from 1.0 to 6.2 and 314 genes with log_2_FC ranged from -1.0 to -5.7). In spheroids challenged with *Borrelia*, 225 genes were significantly upregulated (log_2_FC 1.0 to 5.6) and 396 genes were significantly downregulated (log_2_FC -1.0 to -6.9) (**Figure 4A** and **Supplementary Data Sheet 2**).

298 genes that were upregulated during *Neisseria* infection were downregulated during *Borrelia* infection, whereas 157 genes that were downregulated during *Neisseria* infection were upregulated during *Borrelia* infection. 169 genes were exclusively upregulated during *Neisseria* infection, and during *Borrelia* infection it was 68 genes. The number of genes that were exclusively downregulated during *Neisseria* and *Borrelia* infection was 157 and 97, respectively (**Figure 4B** and **Supplementary Data Sheet 3**).

The Pearsons correlation coefficient (r) of 0.805 and p value = 0.005 between the Log_2_FC values of 10 randomly selected DEGs observed in RNAseq and qRT-PCR validates the differential expression of genes determined by RNAseq (**Supplementary Figure 4**).

Functional annotation of DEGs was performed using the Reactome and DAVID servers, providing categorization of DEGs into various pathways such as cell adhesion, extracellular matrix organization, cytokine signaling, and so on (**Figures 4C, D**; **Supplementary Sata Sheet 4**). In the following part of results, we are presenting the biological process, enriched at BBB that suggests weekended barrier attributes along with the enhanced immune response.

### 3.4 Cell adhesion molecules

**Figure 5:**
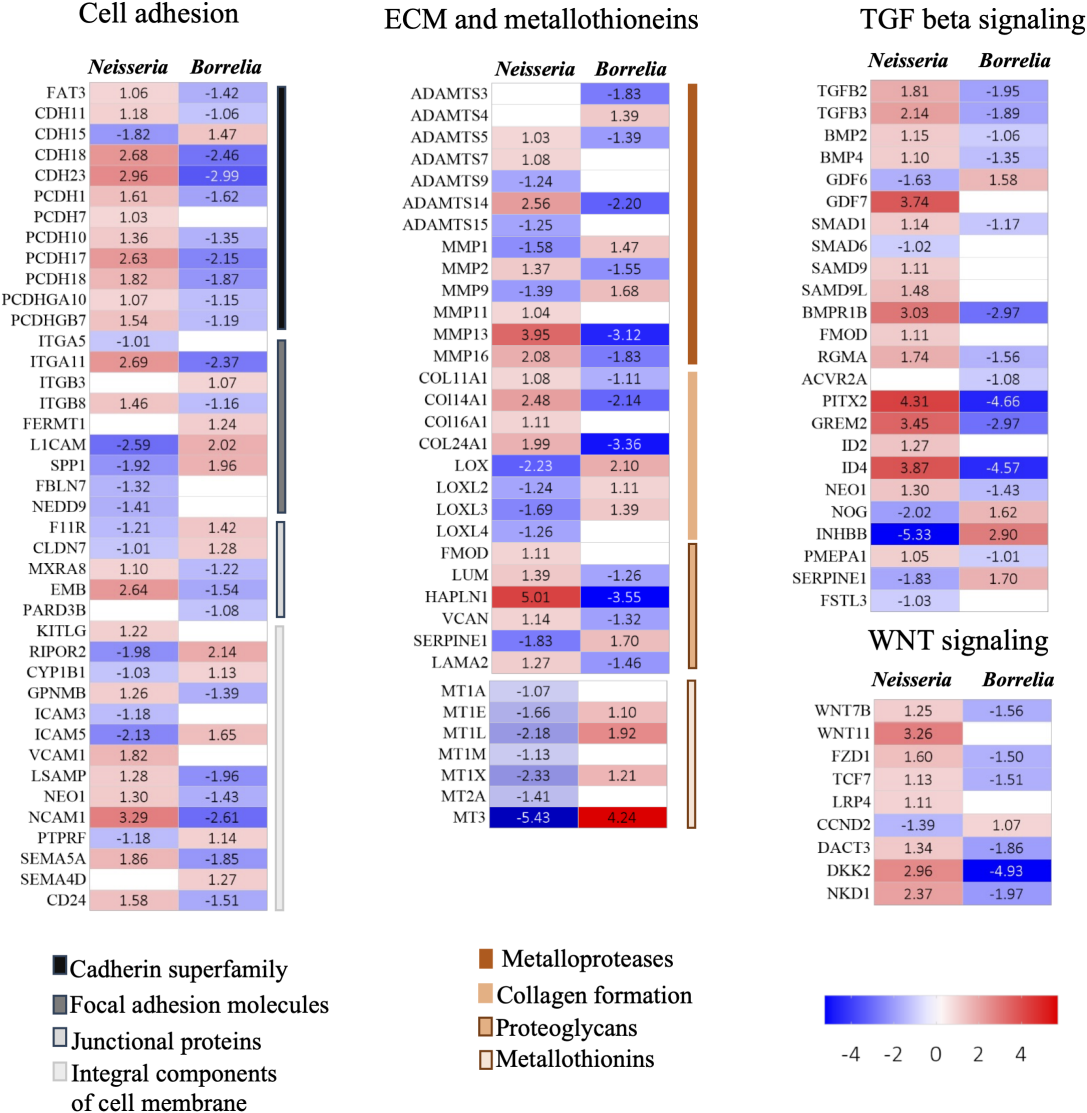
Heatmaps for DEGs. Heatmaps depicting Log_2_FC values of DEGs identified in BBB spheroids undergoing *Neisseria* or *Borrelia* infection. DEGs are categorized into biological functions – cell adhesion, ECM and metallothionins, TGF beta signaling and WNT signaling. Color scale presents Log_2_FC values.

In the infected spheroids, 41 genes belonging to cadherin superfamily, focal adhesion molecules, junctional proteins, and integral components of membrane proteins (all of these pathways govern cell adhesion) were significantly evoked (**Figure 5**; **Supplementary Table 3**). All these genes were inversely evoked in BBB infected with *Neisseria* and *Borrelia*. Genes encoding cadherins (CDHs), protocadherins (PCDHs), and the protocadherin gamma gene cluster - A10 and B7 were upregulated during *Neisseria* infection, whereas the same genes were downregulated during *Borrelia* infection, with an exception of CDH15. Among junctional proteins, the bicellular tight junction genes F11 receptor (F11R) and claudin 7 (CLDN7) were downregulated during *Neisseria* infection but upregulated during *Borrelia* infection (**Figure 5**; **Supplementary Table 3**). Genes encoding integral components of cell membrane (ICAM, VCAM and NCAM-1) were also inversely expressed. However, the pattern of expression for genes related to focal adhesion was not consistent during either of the bacterial infections (**Figure 5**; **Supplementary Table 3**).

Surprisingly, E-selectin’s expression levels remained unchanged in infected spheroids, despite the fact that it was one of the highly expressed DEGs observed previously during *Neisseria* or *Borrelia* infection to hBMECs.

### 3.5 Extracellular matrix related genes and metallothioneins

Several DEGs involved in extracellular matrix reorganization were activated in response to *Neisseria* and *Borrelia* infection (**Figure 5**; **Supplementary Table 3**). Among the metalloproteases, seven ADAMTS (a disintegrin and metalloproteinase with thrombospondin motifs) and six matrix metalloproteinases (MMPs), were differentially expressed. Both ADAMTS and MMPs, are extracellular metalloenzymes that can degrade extracellular matrix (ECM) protein*s*. Besides degrading the components of ECM, MMPs can cleave various, basement membrane proteins, tight junction proteins, cytokines, chemokines and growth factors (Schubert-Unkmeir et al., 2010). Four Collagens (COL11A1, COL14A1, COL16A1, and COL24A1) mediating fibrillogenesis, were upregulated in response to *Neisseria*, but downregulated in during *Borrelia* infection (**Figure 5**; **Supplementary Table 3**). The genes encoding the members of the lysyl oxidase family (Lox, LoxL2, LoxL3 and LoxL4) also showed an opposite pattern of expression during *Neisseria* and *Borrelia* infection. Lysyl oxidases are critical for the crosslinking of elastin and collagen in extracellular matrix during the repair of ECM. Fibromodulin, which interacts with Lox to modulate collagen crosslinking and prevent collaginolysis, was upregulated in *Neisseria* infections. In addition to fibromodulin, *Neisseria* induced the expression of five other ECM-proteoglycans, *viz*., lumican (which stimulates endothelial cell growth and vascular sprouting), hyaluronan, proteoglycan link protein (Hapln-1) and versican (**Figure 5**; **Supplementary Table 3**). Hapln-1 was the only EMC proteoglycan that was downregulated in response to *Borrelia*.

Metallothionins, regulating intracellular zinc and copper trafficking in response to endogenous or exogenous stimuli (such as infection, cytokine release, and oxidative stress), were downregulated during neisserial infection. The list includes - metallothionin 1 (MT1A, MT1E, MT1M, and MT1X), the MT1L pseudogene, and metallothionin 2 and 3. On the other hand four metallothionins - MT1E, MT1L, MT1X, and MT3, were upregulated during *Borrelia* infection (**Figure 5**; **Supplementary Table 3**). In the presence of lipopolysaccharide, metallothionins can aid in the induction of TNF-α and NF-kB, whereas metallothionin knockout causes vacuolar damage in endothelial cell and focal loss of basement membrane. MT2A specifically influences the MMP9 expression and MT3 increases VEGF expression in BMECs (Kanekiyo et al., 2002; Takano et al., 2004).

### 3.6 Signaling by Transforming growth factor beta family members

TGF-β, a multifunctional cytokine, regulates a variety of cellular functions namely cell proliferation, differentiation, adhesion, and migration, all of which are critical for maintaining BBB integrity. There are atleast 30 proteins associated with TGF-β, which form a superfamily, and many of them were differentially expressed in infected spheroids *viz*., ligands of TGF-β (TGFB2, TGFB3, bone morphogenetic protein 2, growth factors - germalin 2, inhibin subunit beta B, and growth differentiation factor 6 and 7), receptors (BMP receptor type 1B, activin A receptor type 2A and neogenin 1), signal transducers (receptor regulated SMADS 1 and 9, and inhibitory SMAD 6) and transcriptional regulators (paired like homeodomain 2(PITX2) and inhibitor of DNA binding 2 and 4) (**Figure 5**; **Supplementary Table 3**). TGF-β signaling occurs in two branches, the TGF-β branch and the BMP branch. Both the branches appeared to be activated during *Neisseria* infection. Three inhibitory molecules were downregulated *viz*. SMAD 6 (Log_2_FC -1.02), noggin (NOG; Log_2_FC -2.02) and inhibin subunit beta b (INHBB; Log_2_FC -5.32). As opposed to *Neisseria* infection, the TGF-β signaling pathway was not activated during borrelial infection as several inhibitory molecules were induced.

### 3.7 WNT signaling pathway

Canonical Wnt/β -catenin signaling pathway can regulate the formation of the BBB (Stenman et al., 2008). In our study, at least 9 DEGs belonging to Wnt/β-catenin signaling are identified, which includes Wnt7b, Wnt11, FZD1, TCF7, LRP4, DKK2, DACT3, NKD1 and CCND2. FZD1 is receptor for Wnt glycoproteins, TCF7 represses transcription of β-catenin target genes, LRP4 is a negative regulator of the Wnt signaling pathway, DACT3 is a dishevelled binding antagonist of β-catenin 3, NKD1 is inhibitor of WNT signaling pathway and CCND2 is an effector of cell cycle progression. Although *Neisseria* infection upregulated Wnt7b, Wnt11, and FZD1, four inhibitors (TCF7, LRP4, DKK2, DACT3 and NKD1) were upregulated and CCND2 which favors cell cycle progression was downregulated. *Borrelia* infection resulted in the inverse expression of the aforementioned genes (**Figure 5**; **Supplementary Table 3**).

### 3.8 Intracellular trafficking

**Figure 6:**
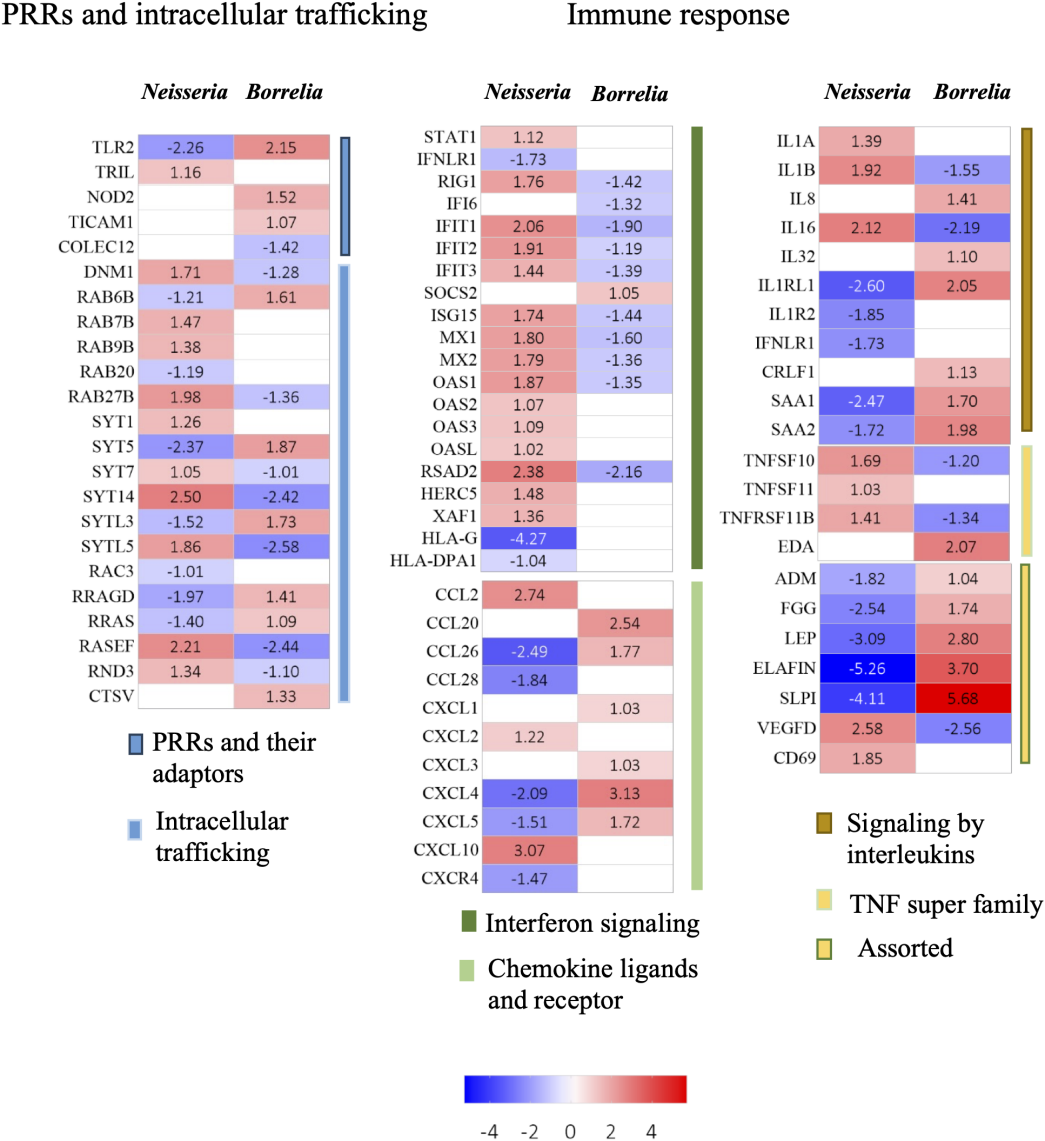
Heatmaps for DEGs. Heatmaps depicting Log_2_FC values of DEGs identified in BBB spheroids undergoing *Neisseria* or *Borrelia* infection. DEGs are categorized into biological functions – PRRS and intracellular trafficking, and immune response. Color scale presents Log_2_FC values.

Eighteen genes associated with endocytosis and intracellular trafficking were differentially expressed in infected spheroids (**Figure 6**; **Supplementary Table 3**). Dynamin 1 (DNM1), a GTPase involved in clathrin-mediated and clathrin-independent endocytic pathways was upregulated during *Neisseria* infection. Surface proteins of *Neisseria* can induce the expression of DNM1 to gain entry into the endothelial cells (Herold et al., 2021). Another GTPase, RAC3 was downregulated only in *Neisseria* infection. Members of RAB GTPse (RAB 6B, 7b, 9b, 20 and 27b) and their effector molecules like synaptotagmin (SYT1, 5, 7, and 14) and exophilins (SYTL 3 and 5) were differentially expressed in infected spheroids that are known to participate in the endocytosis and exocytosis pathways.

### 3.9 Immune response

At least 56 genes differentially evoked in infected spheroids were related to immune response (**Figure 6**; **Supplementary Table 3**).

#### 3.9.1 Pattern recognition receptors

Molecular patterns of both *Neisseria* and *Borrelia* are recognized by members of the family of Toll like receptors, Nod like receptors, C type of lectins, scavenger receptors and integrins (Petnicki-Ocwieja and Kern, 2014) Across all of these receptors and their associated molecules, only five genes changed their expression significantly viz., TLR2, TLR4 interactor with leucine-rich repeats (TRIL4), nucleotide binding oligomerization domain containing 2 (NOD2), TIR domain containing adaptor molecule 1 (TICAM1), and collectin 12 (COLEC12). Surprisingly, the two subunits of NFKB – NFkB1 and NFkB 2 were not differentially expressed in spheroids undergoing *Neisseria* (Log_2_FC 0.61, -0.26) and *Borrelia* (Log_2_FC -0.030, 0.44) infection.

#### 3.9.2 Interleukins and chemokines

In response to infection, four interleukins (IL1A, IL1B, IL8 and IL16), their receptors (IL1 receptor like 1, IL1 receptor 2, IFN-lambda receptor 1, cytokine receptor like factor 1) and two acute phase proteins (Serum amyloid A 1 and 2) were differentially expressed. IL-1A can induce the pro-angiogenic cytokines e.g., CXCL1 in brain endothelial cells, while IL1B can induces expression of C-X-C chemokines, adhesion molecules, and increases vascular permeability (Makó et al., 2010; Dinarello, 2018). Overexpressed IL1A (Log_2_FC 1.39) and IL1B (Log_2_FC 1.92) in spheroids infected with *Neisseria* indicates inflammatory response that could compromise the BBB integrity. In the case of *Borrelia* infection, IL1B was downregulated (Log_2_FC -1.55), however, a proinflammatory cytokine IL32 (Log_2_FC 1.09), a receptor IL1RL1 receptor (Log_2_FC 2.05) and IL8 (Log_2_FC 1.40) were upregulated. The acute phase SAA1 and SAA2 proteins, which also possess cytokine-like properties, had Log_2_FC as 1.70 and 1.98 in *Borrelia* infected spheroids respectively. SAA may stimulate the production and release of matrix metalloproteases (like MMP9 Log_2_FC 1.68), cytokines and chemokines (such as IL8, Log_2_FC 1.69). On the other hand, both SAA1 (Log_2_FC -2.47) and SAA2 (Log_2_FC -1.72) were downregulated during *Neisseria* infection.

Some of members of CC and CXC chemokines were differentially expressed in infected spheroids. Upregulated C-X-C motif chemokine ligand 2 (CXCL2, Log_2_FC 1.21), CXCL10 (Log_2_FC 3.06) and C-C motif chemokine ligand (CCL2, Log_2_FC 2.74) during *Neisseria* infection are important to encourage chemotaxis of neutrophiles, Th1 cells, and monocytes, respectively. Similarly, in spheroids infected with *Borrelia*, upregulated CCL20 (Log_2_FC 2.54), CXCL1 (Log_2_FC 1.02), CXCL3 (Log_2_FC 1.03), CCL26 (Log_2_FC 1.77), CXCL5 (Log_2_FC 1.71), and PF4 (Log_2_FC 3.13) can increase the chemotaxics of dendritic cells, lymphocytes, neutrophils, and eosinophils. Due to the presence of an amphipathic -helix domain or a large patch of positively charged amino acids at the C-terminus, chemokines CXCL10, CCL20, and CCL26 can also act as broad spectrum anti-microbials.

CXCR4 (Log_2_FC -1.46) was the only chemokine receptor downregulated during *Neisseria* infection. As a result, CXCR4 mediated signaling cascade leading to cell proliferation, differentiation, apoptosis and angiogenesis could be hampered during *Neisseria* infection.

#### 3.9.3 Members of TNF superfamily

TNF superfamily members (TNFSF10, 11, 11b) and ectodysplasin A (EDA) were differentially expressed during *Neisseria* or *Borrelia* infection. Upregulation of TNFSF10 (Log_2_FC 1.69) during *Neisseria* infection could promote the induction of JNK or NF-kB mediated inflammatory response or apoptosis. On the other hand, TNFSF10, was downregulated during *Borrelia* infection (Log_2_FC -1.20). TNFSF11 (Log_2_FC 1.03) and TNFRSF11B (Log_2_FC 1.41) upregulated during *Neisseria* infection, could induce CCL20 in astrocytes and promote endothelial cell angiogenesis. In spheroids infected with *Borrelia*, TNFRSF11B was downregulated (Log_2_FC -1.33) and ectodysplasin A (EDA; Log2FC 2.07), was upregulated. Ectodysplasin A can positively regulate the expression of ZO-1 and claudin-1, which is necessary to maintain the barrier function. Whether the upregulated EDA (Log_2_FC 2.07) and overexpressed tight junction proteins – F11R (Log_2_FC 1.42) and CLDN7 (Log_2_FC 1.28) during borrelial infection are interdependent to limit the endothelial barrier disfunction needs further confirmation.

#### 3.9.4 Interferon signaling

Although interferons are produced in response to viral infections, they can also have a limited antibacterial effect, such as inhibiting bacterial migration across endothelial and epithelial barriers (LeMessurier et al., 2013) Prolonged interferon response, on the other hand, can impair cell barrier function (Major et al., 2020). In the present study, signal transducer STAT1, DExD/H-box helicase 58 (RIG1) and several IFN stimulated genes (ISGs) namely IFNα inducible protein 6 (IFI6), Interferon induced protein with tetratricopeptide repeats (IFIT) 1, 2 and 3, ISG15 ubiquitin like modifier (ISG15), HECT and RLD domain containing E3 ubiquitin protein ligase 5 (HERC5), MX dynamin like GTPases (MX 1 and 2), 2’-5’-oligoadenylate synthetase (OAS 1,2 and 3) and OAS-like protein (OASL) were upregulated during *Neisseria* infection and their expression was downregulated during borrelial infection (**Figure 6**; **Supplementary Table 3**). On the other hand, the receptor for type III IFN, Interferon lambda receptor 1 (IFNLR1; Log_2_FC -1.73) was downregulated during *Neisseria* infection which suggests the repression of IFN -λ signaling. The nonclassical human leukocyte antigen G (HLA-G) was strongly downregulated during neisserial infection (Log_2_FC -4.27) (**Figure 6**; **Supplementary Table 3**).

#### 3.9.5 Other important genes

VEGFs, specifically VEGF-A, VEGF-B, VEGF-C, and VEGF-D, as well as their receptors (tyrosin kinase VEGF-R1-3), are important regulators of angiogenesis. VEGF-D expression was found to be upregulated (Log_2_FC 2.58) during *Neisseria* infection but downregulated (Log_2_FC -2.56) during *Borrelia* infection. Because VEGF-D induces the formation of intercellular gaps (Wong et al., 2011), it is possible that the barrier function might be compromised during *Neisseria* infection. Adrenomedullin (ADM) and leptin, both required for endothelial barrier maintenance, were downregulated during *Neisseria* infection (ADM Log_2_FC -1.81, leptin Log2FC -3.08), however their expression levels were increased during Borrelia infection (ADM Log_2_FC 1.04, leptin Log_2_FC 2.80) (**Figure 6**; **Supplementary Table 3**).

## 4 Discussion

A plethora of molecular cross talk occurs between the pathogens and the cells of BBB during its invasion. The Cross talk includes ligand-receptor interactions, cell signaling, host cell responses to resist pathogen invasion, etc. (Kánová et al., 2018; Káňová et al., 2019; Thompson et al., 2020; Tkáčová et al., 2020; Tkáčová et al., 2021). Earlier studies describing the BBB response to invading neuropathogens have focused on BMECs, the barrier’s frontline cells. Nonetheless, it is clear that other cells of the neurovascular unit (pericytes and astrocytes) are equally important in maintaining cell-cell/cell-extracellular matrix interactions and governing the BBB functions. Moreover, studies in the recent past have shown that BBB spheroids are convenient to study brain infections like - cerebral malaria (Adams et al., 2021), fungal meningitis (Kim et al., 2021) and toxoplasmosis (Correa Leite et al., 2021). In the current study, spheroids composed of hBMCEs, pericytes, and astrocytes were used to understand the barrier’s response (via transcriptomic analysis) to neuroinvasive *Neisseria* or *Borrelia* at 3 h post infection in order to capture the events leading to BBB breakdown (**Figures 3E, J**).

Although both *Neisseria* and *Borrelia* are neuroinvasive, they differ significantly in terms of latency time, virulence factors, strategies used to breach the BBB, etc. Capsular polysaccharides, lipo-oligosaccharides, iron-binding proteins, porins, major adhesins (pili, outer-membrane proteins), and phase variable minor adhesins are virulence factors for *Neisseria* (Herold et al., 2019). *Borrelia*, on the other hand, lacks an LPS and iron uptake system but does possess ECM binding, ECM degrading and complement evading lipoproteins that can undergo antigenic variation (Johnson, 2018). While crossing the BBB, *Neisseria* promotes formation of cortical plaques and delocalizes adherence and tight junction proteins (Miller et al., 2013), whereas *Borrelia* activates CD40-mediated signaling cascade and the host’s fibrinolytic system, metalloproteases and proinflammatory cytokines, which may result in fenestrations in hBMECs and transient degradation of tight junction proteins (Grab et al., 2005; Pulzova et al., 2011). Given the differences, we hypothesized that the BBB’s response to both pathogens would differ significantly. The inverse expression of 455 genes in spheroids infected with *Neisseria* or *Borrelia* (**Figure 4D**) supports our assertion.

TLRs, NOD-like receptors, C type lectins, scavenger receptors, integrins, and other PRRs should be activated by *Neisseria* and *Borrelia* (Petnicki-Ocwieja and Kern, 2014; Johswich, 2017). However, only 5 PPR-related genes were found to be differentially expressed (**Figure 6**), namely TLR2, NOD2, and COLEC12 (receptors), TRIL (the modulator of TLR3/4 signaling), and TICAM-1 (the adaptor molecule of TLR3 signaling). In contrast to previous findings (Káňová et al., 2019; Tkáčová et al., 2021), the TLR2 adaptor molecule MyD88 and the transcription regulator NFĸB were not found to be significantly expressed in the current study. The inclusion of pericytes and astrocytes in this study, as opposed to only hBMECs in previous studies, may explain the variation. The same reason could be behind the elevated expression of ICAMs, VCAMS, CD44, E-selectin, and F11R in the monolayer of hBMEC challenged with *Neisseria* (Log_2_FC ≥ 5) (Káňová et al., 2019) as oppose to marginal upregulation of VCAM1 (Log_2_FC 1.82) and rather down regulation of ICAM 3 (Log_2_FC -1.18), ICAM 5 (Log_2_FC -2.13), and F11R (Log_2_FC -1.21) in the present study. Initial colonization of *Neisseria* on hBMECs (**Figure 3B**) may result in abnormal recruitment of polarity complex molecules and adherence junction proteins beneath the bacteria (Miller et al., 2013). Cadherins, ICAMs, and VCAMS, adhesion junction proteins, relocate to the apical regions of hBMECs to form docking structures or transmigratory cups (Doulet et al., 2006). The upregulation of cadherins and VCAM1 in spheroids infected with *Neisseria* may be in response to abnormal translocation (**Figure 5** and **Supplementary Table 3**). No such translocation is expected during *Borrelia* infection as the same cadherin genes were under expressed (**Figure 5** and **Supplementary Table 3**). Tight junction proteins like F11R and CLDN7 are required to maintain barrier function, and their downregulation during *Neisseria* infection may result in transient intercellular junction opening. Despite the fact that F11R and CLDN7 were upregulated during *Borrelia* infection, the other three tight junction proteins, MXRA8, PARD3B, and EMB, were downregulated. Along with the dysregulation of tight junction proteins, focal adhesion molecules like integrins that are required to anchor cells to the extracellular matrix had altered expression. Integrin subunits such as L1CAM, SPP1, and FBLN7, for example, were downregulated during *Neisseria* infection. The downregulation of integrins may have an adverse effect on cell attachment to ECM. Compromised BBB integrity was observed in both *Neisseria* and *Borrelia* infections (**Figures 3E, J**).

Extracellular matrix related protein like MMPs and ADAMTS are secreted in response to the exogenous insults such as invading pathogens. ADAMTS and MMPs can degrade glycocalyx and ECM components, which can result in BBB breakdown, inflammatory cell infiltration, and cytokine signalling dysregulation (Elkington et al., 2005; Muri et al., 2019). In the context of increased permeability of infected spheroids, upregulation of MMPs (MMP 2, 11,13 and 16 in *Neisseria* and MMP1 and 9 in *Borrelia*) and ADAMTS (ADAMTS5, 7 and 14 in *Neisseria* and ADAMTS1, 5 and 14 in Borrelia) is relevant observation. It is also important to note the reduced expression of some of the members of MMP family especially MMP1 and 9 in *Neisseria* and MMP2 and 13 in *Borrelia* infected spheroids. The reduced expression of metalloproteinases during pathogen invasion may be harmful to the host. Other ECM related genes such as collagen synthesizers and lysyl oxidase family members are known to maintain the homeostasis of ECM by stabilizing the elastin and collagen fibres. Additionally they act as chemoattractant for peripheral blood mononuclear cells and inhibit the nuclear translocation of NF-κB (Lucero and Kagan, 2006). It would be interesting to determine if, downregulation of LOX and LOXL genes observed during *Neisseria* infection signals for enhancement in NF-κB dependent inflammatory response at BBB. Since *Borrelia* can damage the collagen and elastic fibres it is necessary to determine if overexpressed LOX and LOXL genes during *Borrelia* infection is host response to damage.

Genes encoding ligands, receptors, signal transducers and transcription factors of TGF-β were mostly upregulated during *Neisseria* infection. However, the same set of genes were downregulated during borrelial infection (**Figure 5**; **Supplementary Table 3**). TGF-β signaling is a context dependent mechanism that can regulate essential biological process such as proliferation and ECM synthesis/degradation (Santibañez et al., 2011). In the context of present study, we predict that TGF-β signaling may have influenced the expression of MMPs as described previously (Schumacher et al., 2023). Several MMPs (at least MMP1, 7, 9, 13 and 14) are known to possess two regulatory domains - TGF-β inhibitory element and Smad binding element (SBE) and therefore, TGF-β can regulate the expression and secretion MMPs. Additionally, TGF-β can activate the transcription factors like AP1, PEA3 and NF*κ*B that can control the MMP expression (Krstic and Santibanez, 2014). In short, the genes involved in TGF signalling are worth investigating in the context of BBB integrity. Another signalling pathway, the Wnt signalling, is important as far as BBB homeostasis is concern. It has been demonstrate that inhibiting Wnt/β-catenin signalling can result in severe defects in brain endothelial cell angiogenesis and breakdown of BBB caused by under expression of tight junction proteins such as claudin (Liebner et al., 2008; Daneman et al., 2009). Several molecules in this pathway including WNT7A, WNT7B, WNT3A, β-catenin, and others, are important in maintaining endothelial cell phenotype (Liebner et al., 2008; Daneman et al., 2009). During *Neisseria* infection, the Wnt signalling pathway appears to be inhibited, as we observed upregulation of 5 inhibitors and downregulation of cycline D2, a cell cycle progression effector molecule (**Figure 5**). It appears that *Neisseria* and *Borrelia* elicit Wnt signaling pathways differently, as the same set of Wnt/β -catenin inhibitors were downregulated during borrelial infection.

Both *Neisseria* and *Borrelia* infections elicited proinflammatory response in spheroids (**Figure 6**) in line with previous findings in BMECs (Káňová et al., 2019; Tkáčová et al., 2021), pericytes (Butsabong et al., 2021; Gil et al., 2022) and astrocytes (Casselli et al., 2017; Geyer et al., 2019). Upregulation of interferon signaling genes (e.g. STAT1, IFIT 1-3 and ISG15, MX 1 - 2 and OAS 1-3 etc), chemokines (eg. CXCL2, CXCL10 and CCL2), interleukins (eg. IL1A, IL1B, IL16) and members of TNF superfamily (TNFSF10 and TNFSF11) can promote secretion of proinflammatory cytokines and MMPs (Kim et al., 2005; Ovstebø et al., 2008; Madhvi et al., 2022), interfere in pathogen replication by either inducing apoptosis or ubiquitin mediated proteasomal degradation (Zhang et al., 2021), induce chemotaxis of leucocytes (Dinarello, 2018) and directly act as antimicrobials (Yang et al., 2003). To contain the bacterial infection, which has a short doubling time (*Neisseria* has only 30 min), an aggravated inflammatory response is essential; however, at the BBB level, such response may compromise barrier function. Along with other proinflammatory genes, elevated levels of VEGFD (Log_2_FC 2.57), IL1B (Log_2_FC 1.92), MMP-2 (Log_2_FC 1.37) and MMP13 (Log_2_FC 3.94) indicate that these molecules may have contributed in increased BBB permeability observed in spheroids. Acute meningitis and altered BBB permeability are common in meningococcal infection patients, but not in neuroborreliosis patients. In spheroids challenged with *Borrelia* interferon related genes (IFIT1-3, IFI6, MX1-2, ISG15) and IL1B were downregulated, while expression of suppressors of cytokine signalling, such as SOCS2, was elevated, which clearly denotes the difference in interferon signalling elicitation by two pathogens (**Figure 6**).

## 5 Conclusion

Self-assembling spheroids comprised of hBMECs, pericytes and astrocytes were cultured to perceive infection of BBB by neuroinvasive *Neisseria* and *Borrelia*. Both the pathogens invaded the BBB spheroids showing adherence with in 1h, followed by massive aggregation until 24 h after exposure. Non-neuroinvasive *E. coli*, on the other hand, did not adhere to the spheroids. The invasion of *Neisseria* and *Borrelia* resulted in complete loss of spheroid integrity within 3 h. The transcriptome analysis revealed underlying molecular events that are responsible for the weakening of the barrier function during *Neisseria* and *Borrelia* infection. Although both *Neisseria* and *Borrelia* increased barrier permeability and altered overall gene expression significantly, 48% of the DEGs were inversely expressed in spheroids infected with *Neisseria* and *Borrelia*. The findings show that, while both *Neisseria* and *Borrelia* can successfully cross the BBB, they elicit the response in the neurovascular unit in very distinct ways. Some of the highly expressed genes (Log_2_ FC > 3.5) in inverse manner during *Neisseria* and *Borrelia* like HAPLN1, MT3, PITX2, ID4, ELAFIN, SLPI, etc., could be targeted to know their relevance in favoring or resisting the infection. Likewise, role of TGF-β and WNT signaling pathways during *Neisseria* and *Borrelia* infection at BBB needs further exploration.

## Data availability statement

The data presented in the study are deposited in the EBI Arrayexpress repository, accession number E-MTAB-13401.

## Ethics statement

Ethical approval was not required for the studies on humans in accordance with the local legislation and institutional requirements because only commercially available established cell lines were used. Ethical approval was not required for the studies on animals in accordance with the local legislation and institutional requirements because only commercially available established cell lines were used.

## Author contributions

AK: Conceptualization, Funding acquisition, Methodology, Visualization, Writing – original draft. JJ: Methodology, Writing – review & editing. KB: Methodology, Writing – review & editing. EM: Funding acquisition, Writing – review & editing. MB: Funding acquisition, Conceptualization, Supervision, Writing – review & editing.

## Supplementary material

Supplementary Image 1 3D confocal images of spheroid showing the localization of three cell types. Cryosection of spheroid used to generate **Figure 1**, panel **E-H** was scanned by LSM700 to depict the localization of prestained hBMEC (red fluorescence), pericytes (green fluorescence) and astrocytes (blue fluroscence). The Z-stack images are arranged in 3D format. It is advised to use ImageJ (https://imagej.net/ij/) for proper visualization of fluorescent signals.

Supplementary Image 2 Confocal microscopy image of spheroid generated by co-culturing prestained cells. Cryosection of spheroid used to generate **Figure 1**, panel **E-H** was scanned by LSM700 to depict the localization of prestained hBMEC (red fluorescence), pericytes (green fluorescence) and astrocytes (blue fluroscence). The Z projections in X-Z directions are shown in top and right panel. The orthogonal planes of the X–Z and Y–Z projection are shown by the green and red lines respectively. It is advised to use ImageJ (https://imagej.net/ij/) for proper visualization of fluorescent signals.
