# Supplementary material for: Differential transcriptome response of blood brain barrier spheroids to neuroinvasive Neisseria and Borrelia
Source: Front Cell Infect Microbiol. 2023 Dec 19;13:1326578. doi: 10.3389/fcimb.2023.1326578 (PMC10766361; doi:10.3389/fcimb.2023.1326578)
Supplement: Supplementary file 6 [file Presentation_1.pdf]

Differential transcriptome response of blood brain barrier spheroids to neuroinvasive *Neisseria* and *Borrelia*

Amod Kulkarni[1,2], Jana Jozefiaková[1], Katarína Bhide[1], Evelína Mochnaćová[1], and Mangesh Bhide[1,2*]

[1]Laboratory of Biomedical Microbiology and Immunology, The University of Veterinary Medicine and Pharmacy, Komenského 73, 04181 Kosice, Slovakia

[2]Institute of Neuroimmunology of Slovak Academy of Sciences, v. v. i., Dúbravská cesta 9, 845 10 Bratislava, Slovakia

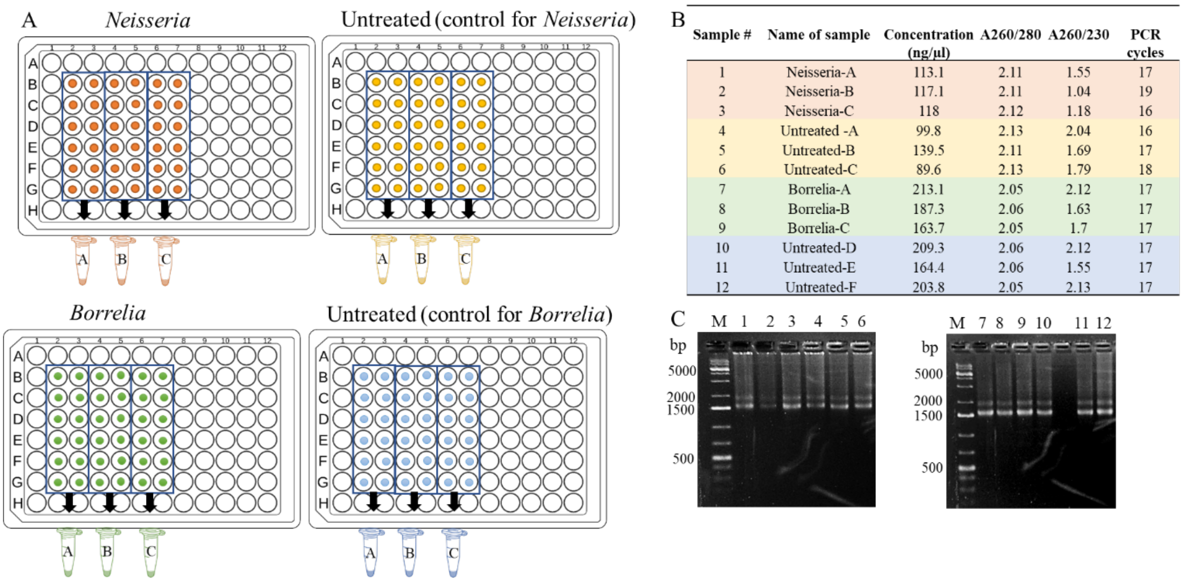

Sample #	Name of sample	Concentration (ng/μl)	A260/280	A260/230	PCR cycles
1	Neisseria-A	113.1	2.11	1.55	17
2	Neisseria-B	117.1	2.11	1.04	19
3	Neisseria-C	118	2.12	1.18	16
4	Untreated -A	99.8	2.13	2.04	16
5	Untreated-B	139.5	2.11	1.69	17
6	Untreated-C	89.6	2.13	1.79	18
7	Borrelia-A	213.1	2.05	2.12	17
8	Borrelia-B	187.3	2.06	1.63	17
9	Borrelia-C	163.7	2.05	1.7	17
10	Untreated-D	209.3	2.06	2.12	17
11	Untreated-E	164.4	2.06	1.55	17
12	Untreated-F	203.8	2.05	2.13	17

Supplementary figure 1: Plan of spheroids used for RNAseq and quality assessment of extracted RNA. (A) Schematic presentation of spheroids designated for RNAseq experiment, pooled replicates per treatment and separate controls for *Neisseria* or *Borrelia* infection are depicted. (B) Concentration of RNA extracted from pooled spheroids exposed to either *Neisseria*, *Borrelia*, or their control groups, purity of extracted RNA(A260/280 and A260/230) measured on nanodrop and number of PCR cycles used to generate cDNA libraries are tabulated. (C) Resolution of RNA extracted from the pooled spheroids on 1% agarose gel is shown. Lane M: 1kb plus DNA ladder, lane 1-12: RNA samples corresponding to samples # 1-12 mentioned in B.

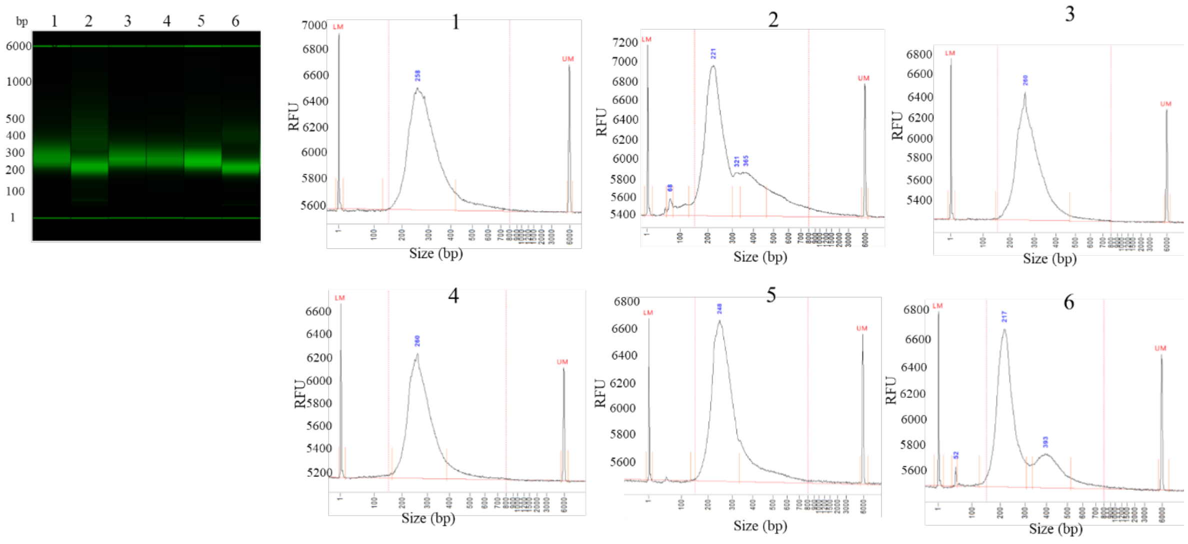

Supplementary figure 2: Quality control of cDNA libraries by fragment analyser

Left figure (Electrophoresis) - Quality control of cDNA libraries by fragment analyser performed prior to sequencing. Lane 1-3: cDNA libraries from spheroids infected with *Neisseria*. Lane 4-6: cDNA libraries from untreated spheroids forming negative control. cDNA libraries were having a fragment size of 150-300 bp. Hence suitable for sequencing.

Graphs on the right hand (panel 1 to 6) - graphical representation of the peaks of DNA fragments derived from electrophoresis. Peaks in panel 1-6 corresponds to cDNA libraries mentioned in Lane 1-6 of the left figure. RFU – relative fluorescence unit, bp –base pair.

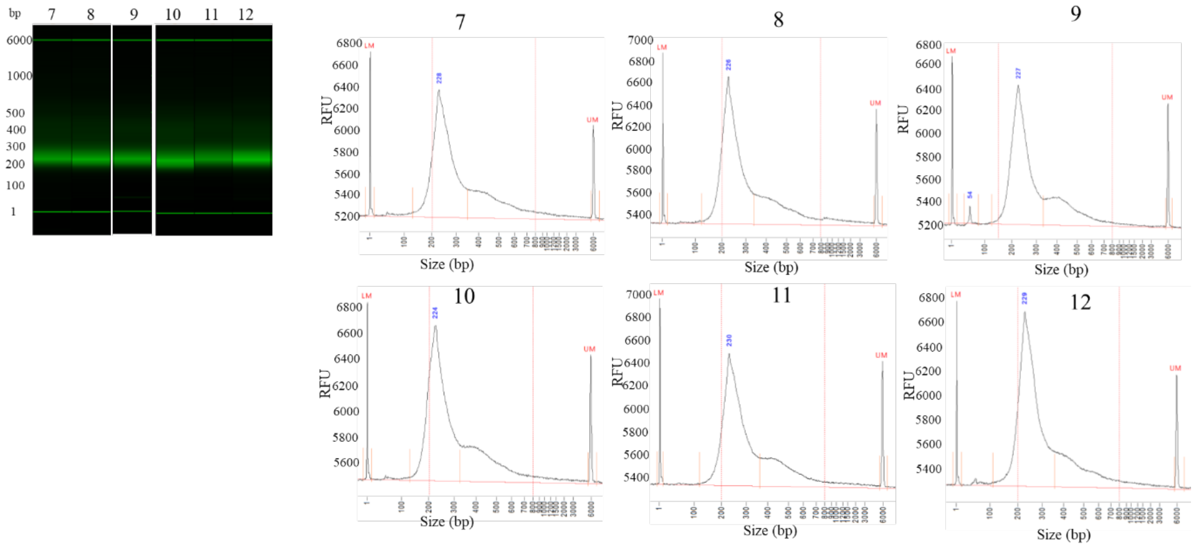

Supplementary figure 3: Quality control of cDNA libraries by fragment analyser

Left figure (Electrophoresis) - Quality control of cDNA libraries by fragment analyser performed prior to sequencing. Lane 7-9: cDNA libraries from spheroids infected with *Borrelia*. Lane 10-12: cDNA libraries from untreated spheroids forming negative control. cDNA libraries were having a fragment size of 150-300 bp. Hence suitable for sequencing.

Graphs on the right hand (panel 7 to 12) - graphical representation of the peaks of DNA fragments derived from electrophoresis. Peaks in panel 7-12 corresponds to cDNA libraries mentioned in Lane 7-12 of the left figure. RFU – relative fluorescence unit, bp –base pair

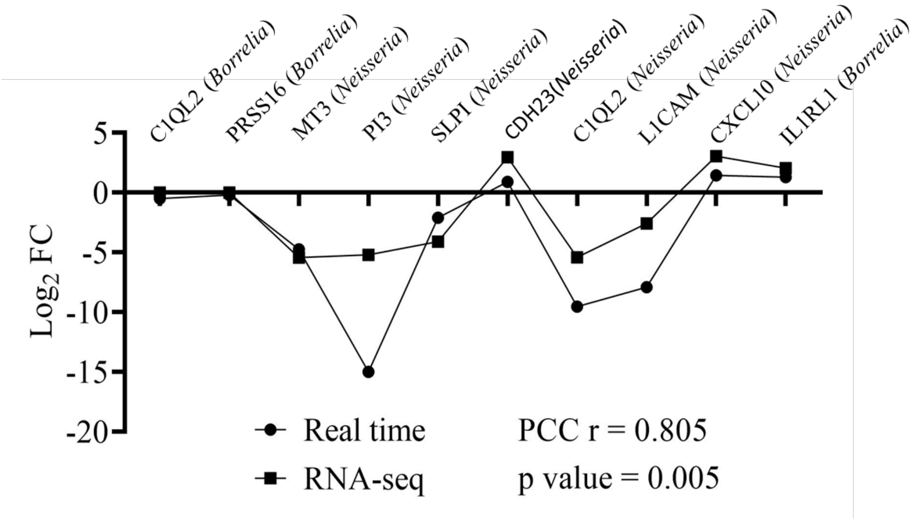

Supplementary figure 4: Validation of RNAseq results using qRT-PCR. Correlation between the Log_2FC values of randomly selected DEGs inRNAseq versus qRT PCR is depicted in line. Abbreviation of selected genes, their Log_2FC values either during *Neisseria* or *Borrelia* infection in parenthesis, Pearsons correlation coefficient (PCC r) and p values are mentioned.

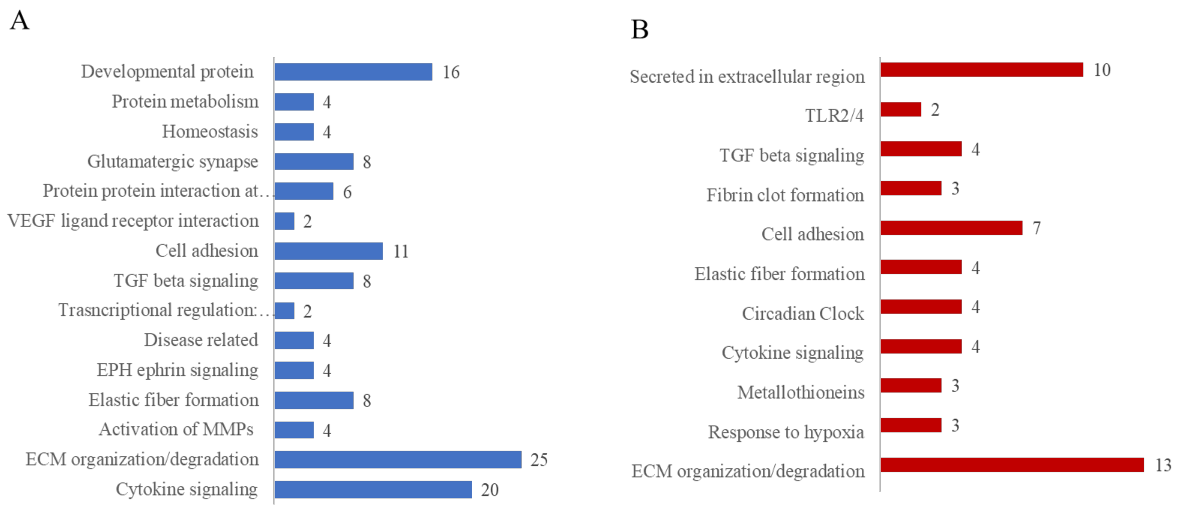

Supplementary figure 5: Commonly expressed DEGs classified into biological pathways (A) commonly expressed DEGs - up regulated during *Neisseria* infection and downregulated during *Borrelia* infection and (B) commonly expressed DEGs - up regulated during *Borrelia* infection and downregulated during *Neisseria* infection were classified into various biological pathways. Number of genes grouped within each biological pathway are denoted above bar graphs.
