# Supplementary material for: Differential transcriptome response of blood brain barrier spheroids to neuroinvasive Neisseria and Borrelia
Source: Front Cell Infect Microbiol. 2023 Dec 19;13:1326578. doi: 10.3389/fcimb.2023.1326578 (PMC10766361; doi:10.3389/fcimb.2023.1326578)
Supplement: Supplementary file 7 [file Table_1.docx]

### Supplementary table 1: Number of raw, preprocessed reads obtained from the RNAseq along and their mapping to human genome

| **Name of cDNA Library** | **Raw reads** | **Preprocessed reads** | **Uniquely mapped reads and percentage (%)** | **Multimapped reads and percentage (%)** | **Uniquely mapped reads assigned to genes and percentage (%)** |
| --- | --- | --- | --- | --- | --- |
| *Neisseria*-A | 9,341,276 | 9,328,563 | 7,352,035 (78.81%) | 1,466,102 (15.72%) | 4,432,641 (54.17%) |
| *Neisseria*-B | 13,998,858 | 13,922,564 | 10,210,225 (73.34%) | 2,137,268 (15.35%) | 5,820,303 (52.02%) |
| *Neisseria*-C | 11,196,776 | 11,180,376 | 8,798,378 (78.69%) | 1,728,166 (15.46%) | 5,358,486 (53.81%) |
| Untreated- A | 10,349,032 | 10,334,696 | 8,098,515 (78.36%) | 1,653,727 (16.00%) | 5,145,373 (55.88%) |
| Untreated-B | 10,503,085 | 10,484,453 | 8,270,545 (78.88%) | 1,624,721 (15.50%) | 5,482,769 (57.11%) |
| Untreated-C | 13,113,511 | 13,055,915 | 9,713,061 (74.40%) | 1,987,543 (15.22%) | 5,262,034 (54.94%) |
| *Borrelia*-A | 9,826,986 | 9,806,054 | 7,756,777 (79.10%) | 1,463,060 (14.92%) | 4,786,448 (56.24%) |
| *Borrelia*-B | 10,977,749 | 10,948,060 | 8,527,732 (77.89%) | 1,637,794 (14.96%) | 5,143,669 (56.10%) |
| *Borrelia*-C | 11,050,077 | 11,022,193 | 8,570,136 (77.75%) | 1,697,593 (15.40% | 5,191,358 (54.29%) |
| Untreated-D | 10,970,959 | 10,938,956 | 8,424,883 (77.02%) | 1,605,299 (14.68%) | 5,208,747 (53.38) |
| Untreated-E | 11,629,203 | 11,598,124 | 9,151,538 (78.91%) | 1,692,395 (14.59%) | 5,143,924 (56.03%) |
| Untreated-F | 11,016,322 | 10,984,726 | 8,573,474 (78.05%) | 1,702,979 (15.50) | 5,465,883 (53.25%) |
