# Supplementary material for: Differential transcriptome response of blood brain barrier spheroids to neuroinvasive Neisseria and Borrelia
Source: Front Cell Infect Microbiol. 2023 Dec 19;13:1326578. doi: 10.3389/fcimb.2023.1326578 (PMC10766361; doi:10.3389/fcimb.2023.1326578)
Supplement: Supplementary file 8 [file Table_2.docx]

| **Supplementary Table 2**. **Primers used in real-time PCR** | | | | |
| --- | --- | --- | --- | --- |
| **S.No.** | **Gene name** | **Primer name** | **Sequence (5’ to 3’)** | **Amplicon size (bp)** |
| 1 | Complement C1q like 2 | C1QL2_F | TCTTCACCTACCACATCCTCAT | 212 |
|  |  | C1QL2_ R | TAGTCGTAGTTCTGGTCGGC |  |
| 2 | Serine protease 16 | PRSS16_F | TGGGGCTAACAAAGTGCTGT | 137 |
|  |  | PRSS16_R | TCTCAGGTGCCATGTCCAAG |  |
| 3 | metallothionein 3 | MT3_ F | CGTCCAGTTGCTTGGAGAAG | 134 |
|  |  | MT3_ R | ATTTGCATCCCTCGCACTTG |  |
| 4 | Peptidase inhibitor 3 | PI3_ F | CCTGTTTCGTTCCCCAGTGA | 124 |
|  |  | PI3_R | GGAAGAATGGACAGTGTGGGA |  |
| 5 | Secretory leukocyte peptidase inhibitor | SLPI_ F | AAATCCTGCGTTTCCCCTGT | 102 |
|  |  | SLPI_R | GCCAAGTCTCAGGGTGGAAA |  |
| 6 | cadherin related 23 | CDH23_F | TTGATGGAGCCAACCCTGTG | 148 |
|  |  | CDH23_R | CCAAAAGTTCCATGGGTGCG |  |
| 7 | L1 cell adhesion molecule | L1CAM_ F | GGATGTTCCGGCACCAAATG | 127 |
|  |  | L1CAM_R | ACGAGGAGCAGGAGGATGAT |  |
| 8 | C-X-C motif chemokine ligand 10 | CXCL10_F | GCCATTCTGATTTGCTGCCTT | 179 |
|  |  | CXCL10_R | GCAATGATCTCAACACGTGGAC |  |
| 9 | Interleukin 1 receptor like 1 | IL1RL1_F | AAGGTACAGGGCGCACAAG | 130 |
|  |  | IL1RL1_R | CCTTGCTCATCCTTGACCGT |  |
| 10 | β2-microtubulin (Reference gene) | b2m_F | GCTCGCGCTACTCTCTCTTT | 55 |
|  |  | b2m_R | CGGATGGATGAAACCCAGACA |  |
| 11 | Glyceraldehyde 3-phosphate dehydrogenase  (Reference gene) | GAPDH_F | TCACCAGGGCTGCTTTTAACT | 118 |
|  |  | GAPDH_R | TGACAAGCTTCCCGTTCTCAG |  |
