# Supplementary material for: Differential transcriptome response of blood brain barrier spheroids to neuroinvasive Neisseria and Borrelia
Source: Front Cell Infect Microbiol. 2023 Dec 19;13:1326578. doi: 10.3389/fcimb.2023.1326578 (PMC10766361; doi:10.3389/fcimb.2023.1326578)
Supplement: Supplementary file 9 [file Table_3.docx]

**Supplementary Table 3: Log2 fold change (Log_2_FC) values of differentially expressed gene (DEGs) categorized into various biological functions**

PRRs and intracellular trafficking

| Cluster | Ensembl ID | Gene Name | Log_2_FC in *Neisseria* | Log_2_FC in *Borrelia* |
| --- | --- | --- | --- | --- |
| PRRs and their adaptors | ENSG00000137462 | Toll like receptor 2 (TLR2) | -2.2649 | 2.153731 |
|  | ENSG00000255690 | TLR4 interactor with leucine-rich repeats (TRIL) | 1.155055 | NS |
|  | ENSG00000167207 | NOD2 | NS | 1.518683 |
|  | ENSG00000127666 | TIR Domain Containing Adaptor Molecule 1 (TICAM1) | NS | 1.074043 |
|  | ENSG00000158270 | Collectin 12 (COLEC12) | NS | -1.42172 |
| intracellular trafficking | ENSG00000106976 | Dynamin 1 (DNM1) | 1.710094 | -1.2834 |
|  | ENSG00000154917 | RAB6B | -1.20882 | 1.60694 |
|  | ENSG00000276600 | RAB7B | 1.473278 | NS |
|  | ENSG00000123570 | RAB9B | 1.380581 | NS |
|  | ENSG00000139832 | RAB20 | -1.18968 | NS |
|  | ENSG00000041353 | RAB27B | 1.981687 | -1.36039 |
|  | ENSG00000067715 | Synaptotagmin 1 (SYT1) | 1.262252 | NS |
|  | ENSG00000129990 | Synaptotagmin 5 (SYT5) | -2.36789 | 1.872914 |
|  | ENSG00000011347 | Synaptotagmin 7 (SYT7) | 1.0453 | -1.01334 |
|  | ENSG00000143469 | Synaptotagmin 14 (SYT14) | 2.502689 | -2.42141 |
|  | ENSG00000164674 | Synaptotagmin like 3 (SYTL3) | -1.52405 | 1.727451 |
|  | ENSG00000147041 | Synaptotagmin like 5 (SYTL5) | 1.858076 | -2.57662 |
|  | ENSG00000169750 | Rac family small GTPase 3 (RAC3) | -1.01446 | NS |
|  | ENSG00000025039 | Ras related GTP binding D (RRAGD) | -1.9697 | 1.414861 |
|  | ENSG00000126458 | RAS related (RRAS) | -1.39722 | 1.094984 |
|  | ENSG00000165105 | RAS and EF-hand domain containing (RASEF) | 2.209154 | -2.44111 |
|  | ENSG00000115963 | Rho family GTPase 3 (RND3) | 1.344687 | -1.10187 |
|  | ENSG00000136943 | Cathepsin V | NS | 1.334536 |

DEGs belonging to cell adhesion

| Cluster | ID | Gene Name | Log_2_FC in *Neisseria* | Log_2_FC in *Borrelia* |
| --- | --- | --- | --- | --- |
| Cadherin superfamily | ENSG00000165323 | FAT atypical cadherin 3 (FAT3) | 1.060379 | -1.41905 |
|  | ENSG00000140937 | Cadherin 11 (CDH11) | 1.184009 | -1.05625 |
|  | ENSG00000129910 | Cadherin 15 (CDH15) | -1.82142 | 1.473792 |
|  | ENSG00000145526 | Cadherin 18 (CDH18) | 2.675475 | -2.46029 |
|  | ENSG00000107736 | Cadherin related 23 (CDH23) | 2.961021 | -2.98689 |
|  | ENSG00000156453 | Protocadherin 1 (PCDH1) | 1.611311 | -1.61847 |
|  | ENSG00000169851 | Protocadherin 7 (PCDH7) | 1.027854 | NS |
|  | ENSG00000138650 | Protocadherin 10 (PCDH10) | 1.355202 | -1.35207 |
|  | ENSG00000118946 | Protocadherin 17 (PCDH17) | 2.630041 | -2.15176 |
|  | ENSG00000189184 | Protocadherin 18 (PCDH18) | 1.815012 | -1.8672 |
|  | ENSG00000253846 | Protocadherin gamma subfamily A, 10 (PCDHGA10) | 1.073057 | -1.15407 |
|  | ENSG00000254122 | Protocadherin gamma subfamily B, 7 (PCDHGB7) | 1.537212 | -1.18723 |
| Focal adhesion molecules | ENSG00000161638 | Integrin subunit alpha 5 (ITGA5) | -1.01138 | NS |
|  | ENSG00000137809 | Integrin subunit alpha 11 (ITGA11) | 2.690551 | -2.36726 |
|  | ENSG00000259207 | Integrin subunit beta 3 (ITGB3) | NS | 1.065837 |
|  | ENSG00000105855 | Integrin subunit beta 8 (ITGB8) | 1.457642 | -1.16382 |
|  | ENSG00000101311 | FERM domain containing kindlin 1 (FERMT1) | NS | 1.244012 |
|  | ENSG00000198910 | L1 cell adhesion molecule (L1CAM) | -2.58673 | 2.024099 |
|  | ENSG00000118785 | Secreted phosphoprotein 1 (SPP1) | -1.91685 | 1.955074 |
|  | ENSG00000144152 | Fibulin 7 (FBLN7) | -1.31609 | NS |
|  | ENSG00000111859 | Neural precursor cell expressed, and developmentally down-regulated 9 (NEDD9) | -1.40582 | NS |
| Junctional proteins | ENSG00000158769 | F11 receptor (F11R) | -1.21194 | 1.421156 |
|  | ENSG00000181885 | Claudin 7(CLDN7) | -1.00693 | 1.277694 |
|  | ENSG00000162576 | Matrix remodeling associated 8 (MXRA8) | 1.101426 | -1.22277 |
|  | ENSG00000170571 | Embigin (EMB) | 2.635453 | -1.53846 |
|  | ENSG00000116117 | Par-3 family cell polarity regulator beta (PARD3B) | NS | -1.07651 |
| Integral components of cell membrane | ENSG00000049130 | KIT ligand (KITLG) | 1.21761 | NS |
|  | ENSG00000111913 | RHO family interacting cell polarization regulator 2 (RIPOR2) | -1.97792 | 2.135451 |
|  | ENSG00000138061 | Cytochrome P450 family 1 subfamily B member 1 (CYP1B1) | -1.03465 | 1.13451 |
|  | ENSG00000136235 | Glycoprotein nmb (GPNMB) | 1.255165 | -1.39043 |
|  | ENSG00000076662 | Intercellular adhesion molecule 3 (ICAM3) | -1.18271 | NS |
|  | ENSG00000105376 | Intercellular adhesion molecule 5 (ICAM5) | -2.12817 | 1.649909 |
|  | ENSG00000162692 | Vascular cell adhesion molecule 1(VCAM1) | 1.822444 | NS |
|  | ENSG00000185565 | Limbic system associated membrane protein (LSAMP) | 1.281747 | -1.9612 |
|  | ENSG00000067141 | Neogenin 1 (NEO1) | 1.30478 | -1.42925 |
|  | ENSG00000149294 | Neural cell adhesion molecule 1 (NCAM1) | 3.291043 | -2.60825 |
|  | ENSG00000142949 | Protein tyrosine phosphatase receptor type F (PTPRF) | -1.17894 | 1.143878 |
|  | ENSG00000112902 | Semaphorin 5A (SEMA5A) | 1.859208 | -1.84509 |
|  | ENSG00000187764 | Semaphorin 4D (SEMA4D) | NS | 1.267812 |
|  | ENSG00000272398 | CD24 | 1.581121 | -1.50738 |

DEGs belonging to extracellular matrix

| Cluster | ID | Gene Name | Log_2_FC in *Neisseria* | Log_2_FC in *Borrelia* |
| --- | --- | --- | --- | --- |
| Metallo-proteases | ENSG00000156140 | ADAM metallopeptidase with thrombospondin type 1 motif 3 (ADAMTS3) | NS | -1.8286 |
|  | ENSG00000158859 | ADAM metallopeptidase with thrombospondin type 1 motif 4 (ADAMTS4) | NS | 1.386571 |
|  | ENSG00000154736 | ADAM metallopeptidase with thrombospondin type 1 motif 5 (ADAMTS5) | 1.031168 | -1.38669 |
|  | ENSG00000136378 | ADAM metallopeptidase with thrombospondin type 1 motif 7 (ADAMTS7) | 1.077029 | NS |
|  | ENSG00000163638 | ADAM metallopeptidase with thrombospondin type 1 motif 9 (ADAMTS9) | -1.23737 | NS |
|  | ENSG00000138316 | ADAM metallopeptidase with thrombospondin type 1 motif 14 (ADAMTS14) | 2.555103 | -2.19888 |
|  | ENSG00000166106 | ADAM metallopeptidase with thrombospondin type 1 motif 15 (ADAMTS15) | -1.25321 | NS |
|  | ENSG00000196611 | Matrix metallopeptidase 1 (MMP1) | -1.58344 | 1.468941 |
|  | ENSG00000087245 | Matrix metallopeptidase 2 (MMP2) | 1.372992 | -1.55133 |
|  | ENSG00000100985 | Matrix metallopeptidase 9 (MMP9) | -1.39121 | 1.684208 |
|  | ENSG00000099953 | Matrix metallopeptidase 11 (MMP11) | 1.041023 | NS |
|  | ENSG00000137745 | Matrix metallopeptidase 13 (MMP13) | 3.945848 | -3.12461 |
|  | ENSG00000156103 | Matrix metallopeptidase 16 (MMP16) | 2.07588 | -1.82685 |
| Collagen formation | ENSG00000060718 | Collagen type XI alpha 1 chain (CoL11A1) | 1.076032 | -1.11077 |
|  | ENSG00000187955 | Collagen type XIV alpha 1 Chain (Col14A1) | 2.482196 | -2.1413 |
|  | ENSG00000084636 | Collagen type XVI alpha 1 Chain (Col16A1) | 1.112625 | NS |
|  | ENSG00000171502 | Collagen type XXIV alpha 1 Chain (COL24A1) | 1.99007 | -3.3614 |
|  | ENSG00000113083 | Lysyl Oxidase (LOX) | -2.23177 | 2.103486 |
|  | ENSG00000134013 | Lysyl Oxidase Like 2 (LoxL2) | -1.24267 | 1.110797 |
|  | ENSG00000115318 | Lysyl Oxidase Like 3 (LoxL3) | -1.68781 | 1.394417 |
|  | ENSG00000138131 | Lysyl Oxidase Like 4 (LOXL4) | -1.2624 | NS |
| ECM proteoglycans | ENSG00000122176 | Fibromodulin (FMOD) | 1.109423 | NS |
|  | ENSG00000139329 | Lumican (LUM) | 1.394044 | -1.25582 |
|  | ENSG00000145681 | Hyaluronan and proteoglycan link protein 1 (HAPLN1) | 5.013212 | -3.54751 |
|  | ENSG00000038427 | Versican (VCAN) | 1.141173 | -1.31775 |
|  | ENSG00000106366 | Serpin family E member 1 (SERPINE1) | -1.826 | 1.700109 |
|  | ENSG00000196569 | Laminin subunit alpha 2 (LAMA2) | 1.268658 | -1.45873 |

DEGs belonging to Metallothionins

| Cluster | ID | Gene Name | Log_2_FC in *Neisseria* | Log_2_FC in *Borrelia* |
| --- | --- | --- | --- | --- |
| Metallo-thioneins | ENSG00000205362 | Metallothionein 1A (MT1A) | -1.06751 | NS |
|  | ENSG00000169715 | Metallothionein 1E (MT1E) | -1.66274 | 1.0974 |
|  | ENSG00000260549 | Metallothionein 1L, pseudogene (MT1L) | -2.18415 | 1.917687 |
|  | ENSG00000205364 | Metallothionein 1M (MT1M) | -1.12817 | NS |
|  | ENSG00000187193 | Metallothionein 1X (MT1X) | -2.33377 | 1.213748 |
|  | ENSG00000125148 | Metallothionein 2A (MT2A) | -1.40819 | NS |
|  | ENSG00000087250 | Metallothionein 3 (MT3) | -5.42923 | 4.244263 |

DEGs belonging to TGF beta signaling pathway

| Cluster | ID | Gene Name | Log_2_FC in *Neisseria* | Log_2_FC in *Borrelia* |
| --- | --- | --- | --- | --- |
| Signaling by TGF Beta family members | ENSG00000092969 | Transforming growth factor beta 2 (TGFB2) | 1.814279 | -1.94714 |
|  | ENSG00000119699 | Transforming growth factor beta 3 (TGFB3) | 2.139682 | -1.88708 |
|  | ENSG00000125845 | Bone morphogenetic protein 2 (BMP2) | 1.149144 | -1.06091 |
|  | ENSG00000125378 | Bone morphogenetic protein 4 (BMP4) | 1.102074 | -1.3465 |
|  | ENSG00000156466 | Growth differentiation factor 6(GDF6) | -1.63415 | 1.577524 |
|  | ENSG00000143869 | Growth differentiation factor 7(GDF7) | 3.736373 | NS |
|  | ENSG00000170365 | SMAD family member 1 (SMAD1) | 1.140376 | -1.16685 |
|  | ENSG00000137834 | SMAD family member 6 (SMAD6) | -1.02307 | NS |
|  | ENSG00000205413 | Sterile alpha motif domain containing 9 (SAMD9) | 1.114139 | NS |
|  | ENSG00000177409 | Sterile alpha motif domain containing 9 like (SAMD9L) | 1.483861 | NS |
|  | ENSG00000138696 | Bone morphogenetic protein receptor type 1B (BMPR1B) | 3.033147 | -2.97251 |
|  | ENSG00000122176 | Fibromodulin (FMOD) | 1.109423 | NS |
|  | ENSG00000182175 | Repulsive guidance molecule BMP co-receptor a (RGMA) | 1.739366 | -1.55769 |
|  | ENSG00000121989 | Activin A receptor type 2A (ACVR2A) | NS | -1.08266 |
|  | ENSG00000164093 | Paired like homeodomain 2(PITX2) | 4.307407 | -4.66234 |
|  | ENSG00000180875 | Gremlin 2 (GREM2) | 3.452468 | -2.96984 |
|  | ENSG00000115738 | Inhibitor of DNA binding 2(ID2) | 1.26944 | NS |
|  | ENSG00000172201 | Inhibitor of DNA binding 4, HLH protein (ID4) | 3.871053 | -4.5655 |
|  | ENSG00000067141 | Neogenin 1 (NEO1) | 1.30478 | -1.42925 |
|  | ENSG00000183691 | Noggin (NOG) | -2.02275 | 1.61861 |
|  | ENSG00000163083 | Inhibin subunit beta B (INHBB) | -5.32958 | 2.90129 |
|  | ENSG00000124225 | Prostate transmembrane protein, androgen induced 1 (PMEPA1) | 1.04896 | -1.0147 |
|  | ENSG00000106366 | Serpin family E member 1 (SERPINE1) | -1.82582 | 1.700109 |
|  | ENSG00000070404 | Follistatin like 3 (FSTL3) | -1.03333 | NS |

DEGs belonging to WNT signaling pathway

| Cluster | ID | Gene Name | Log_2_FC in *Neisseria* | Log_2_FC in *Borrelia* |
| --- | --- | --- | --- | --- |
| WNT signaling pathway | ENSG00000188064 | Wnt family member 7b (WNT7B) | 1.246998 | -1.56429 |
|  | ENSG00000085741 | Wnt family member 11 (WNT11) | 3.261576 | NS |
|  | ENSG00000157240 | Frizzled class receptor 1 (FZD1) | 1.600789 | -1.5042 |
|  | ENSG00000081059 | Transcription factor 7 (TCF7) | 1.129927 | -1.51045 |
|  | ENSG00000134569 | LDL receptor related protein 4 (LRP4) | 1.113105 | NS |
|  | ENSG00000118971 | Cyclin D2 (CCND2) | -1.39076 | 1.06899 |
|  | ENSG00000197380 | Dishevelled binding antagonist of beta catenin 3 (DACT3) | 1.335923 | -1.86241 |
|  | ENSG00000155011 | Dickkopf WNT signaling pathway inhibitor 2 (DKK2) | 2.957831 | -4.93076 |
|  | ENSG00000140807 | NKD inhibitor of WNT signaling pathway 1 (NKD1) | 2.368396 | -1.97151 |

DEGs related to immune response

| Cluster | ID | Gene Name | Log_2_FC in *Neisseria* | Log_2_FC in *Borrelia* |
| --- | --- | --- | --- | --- |
| Interferon signaling | ENSG00000115415 | Signal transducer and activator of transcription 1 (STAT1) | 1.117197 | NS |
|  | ENSG00000185436 | Interferon lambda receptor 1 (IFNLR1) | -1.72960 | NS |
|  | ENSG00000107201 | DExD/H-box helicase 58 (RIG1) | 1.762775 | -1.42228 |
|  | ENSG00000126709 | Interferon alpha inducible protein (IFI6) | NS | -1.31765 |
|  | ENSG00000185745 | Interferon induced protein with tetratricopeptide repeats 1 (IFIT1) | 2.06123 | -1.90096 |
|  | ENSG00000119922 | Interferon induced protein with tetratricopeptide repeats (IFIT2) | 1.91237 | -1.19326 |
|  | ENSG00000119917 | Interferon induced protein with tetratricopeptide repeats (IFIT3) | 1.44264 | -1.3864 |
|  | ENSG00000120833 | Suppressor of cytokine signaling 2 (SOCS2) | NS | 1.053633 |
|  | ENSG00000187608 | ISG15 ubiquitin like modifier (ISG15) | 1.73730 | -1.44166 |
|  | ENSG00000157601 | MX dynamin like GTPase 1 (MX1) | 1.79734 | -1.60101 |
|  | ENSG00000183486 | MX dynamin like GTPase 2 (MX2) | 1.78806 | -1.36123 |
|  | ENSG00000089127 | 2'-5'-oligoadenylate synthetase 1 (OAS1) | 1.87263 | -1.35027 |
|  | ENSG00000111335 | 2'-5'-oligoadenylate synthetase 2 (OAS2) | 1.07430 | NS |
|  | ENSG00000111331 | 2'-5'-oligoadenylate synthetase 3 (OAS3) | 1.09479 | NS |
|  | ENSG00000135114 | 2'-5'-oligoadenylate synthetase like (OASL) | 1.02108 | NS |
|  | ENSG00000134321 | Radical S-adenosyl methionine domain containing (RSAD2) | 2.38490 | -2.16258 |
|  | ENSG00000138646 | HECT and RLD domain containing E3 ubiquitin protein ligase 5 (HERC5) | 1.48318 | NS |
|  | ENSG00000132530 | XIAP associated factor 1 (XAF1) | 1.35628 | NS |
|  | ENSG00000204632 | Non-classical human leukocyte antigen G (HLA-G) | -4.2713 | NS |
|  | ENSG00000231389 | MHC, class II, DP alpha 1 (HLA-DPA1) | -1.0409 | NS |
| Chemokine receptor bind chemokine | ENSG00000108691 | C-C motif chemokine ligand 2 (CCL2/MCP1) | 2.742216 | NS |
|  | ENSG00000115009 | C-C motif chemokine ligand 20 (CCL20/MIP3A) | NS | 2.544549 |
|  | ENSG00000006606 | C-C motif chemokine ligand (CCL26 MIP4A) | -2.4894 | 1.772875 |
|  | ENSG00000151882 | C-C motif chemokine ligand 28 (CCL28) | -1.8444 | NS |
|  | ENSG00000163739 | C-X-C motif chemokine ligand 1 (CXCL1) | NS | 1.027137 |
|  | ENSG00000081041 | C-X-C motif chemokine ligand 2 (CXCL2/MIP2A) | 1.21665 | NS |
|  | ENSG00000163734 | C-X-C motif chemokine ligand 3 (CXCL3/MIP2B) | NS | 1.031078 |
|  | ENSG00000163737 | Platelet factor 4 (PF4/CXCL4) | -2.0886 | 3.130214 |
|  | ENSG00000163735 | C-X-C motif chemokine ligand (CXCL5) | -1.5059 | 1.717715 |
|  | ENSG00000169245 | C-X-C motif chemokine ligand 10 (CXCL10) | 3.066795 | NS |
|  | ENSG00000121966 | C-X-C motif chemokine receptor 4 (CXCR4) | -1.4670 | NS |
| Signaling by interleukins | ENSG00000115008 | Interleukin 1 alpha (IL1A) | 1.39002 | NS |
|  | ENSG00000125538 | Interleukin 1 beta (IL1B) | 1.92217 | -1.54609 |
|  | ENSG00000169429 | Interleukin 8 (IL8) | NS | 1.406147 |
|  | ENSG00000172349 | Interleukin-16 (IL16) | 2.12268 | -2.19327 |
|  | ENSG00000008517 | Interleukin 32 (IL32) | NS | 1.096394 |
|  | ENSG00000115602 | Interleukin 1 receptor like 1 (IL1RL1) | -2.6036 | 2.053328 |
|  | ENSG00000115590 | Interleukin 1 receptor type 2 (IL1R2) | -1.8505 | NS |
|  | ENSG00000185436 | Interferon lambda receptor 1 (IFNLR1) | -1.7296 | NS |
|  | ENSG00000006016 | Cytokine receptor like factor (CRLF1) | NS | 1.125765 |
|  | ENSG00000173432 | Serum amyloid A1 (SAA1) | -2.4715 | 1.696785 |
|  | ENSG00000134339 | Serum amyloid A2 (SAA2) | -1.7230 | 1.982181 |
| TNF super family | ENSG00000121858 | TNF superfamily member 10 (TNFSF10/TRAIL) | 1.68905 | -1.19808 |
|  | ENSG00000120659 | TNF superfamily member 11 (TNFSF11/RANKL) | 1.02687 | NS |
|  | ENSG00000164761 | TNF receptor superfamily member 11b (TNFRSF11B/OPG) | 1.40620 | -1.3378 |
|  | ENSG00000158813 | Ectodysplasin A (EDA) | NS | 2.072629 |
| Assorted | ENSG00000148926 | Adrenomedullin (ADM) | -1.8189 | 1.036383 |
|  | ENSG00000171557 | Fibrinogen gamma chain (FGG) | -2.5433 | 1.739595 |
|  | ENSG00000174697 | Leptin (LEP) | -3.0857 | 2.795976 |
|  | ENSG00000124102 | peptidase inhibitor 3 (Elafin) | -5.2608 | 3.697057 |
|  | ENSG00000124107 | Secretory leukocyte peptidase inhibitor (SLPI) | -4.1059 | 5.67797 |
|  | ENSG00000165197 | Vascular endothelial growth factor D (VEGFD) | 2.57819 | -2.56110 |
|  | ENSG00000110848 | CD69 /CLEC2C | 1.85348 | NS |
