# Supplementary figures and images for: Differential transcriptome response of blood brain barrier spheroids to neuroinvasive Neisseria and Borrelia
Source: Front Cell Infect Microbiol. 2023 Dec 19;13:1326578. doi: 10.3389/fcimb.2023.1326578 (PMC10766361; doi:10.3389/fcimb.2023.1326578)

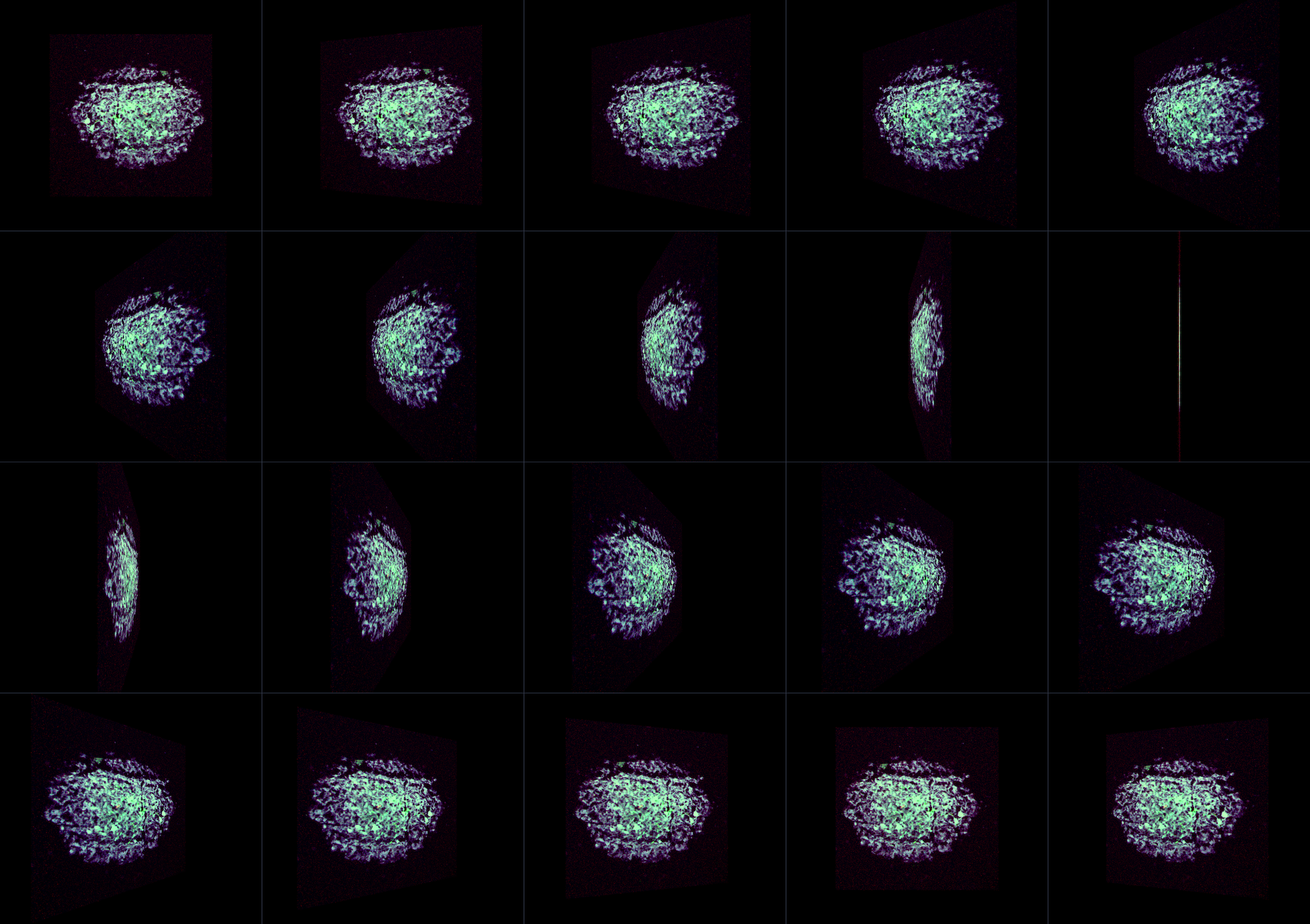

Supplement: Supplementary Image 1 — 3D confocal images of spheroid showing the localization of three cell types. Cryosection of spheroid used to generate Figure 1, panel E-H was scanned by LSM700 to depict the localization of prestained hBMEC (red fluorescence), pericytes (green fluorescence) and astrocytes (blue fluroscence). The Z-stack images are arranged in 3D format. It is advised to use ImageJ (https://imagej.net/ij/) for proper visualization of fluorescent signals. [file Image_1.tif]

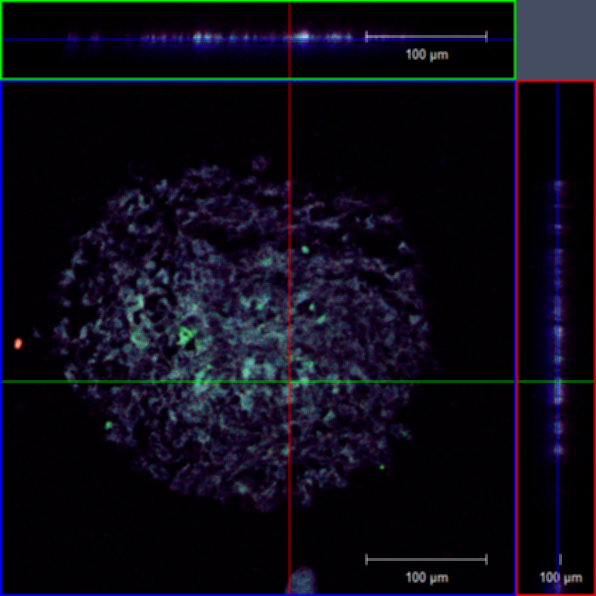

Supplement: Supplementary Image 2 — Confocal microscopy image of spheroid generated by co-culturing prestained cells. Cryosection of spheroid used to generate Figure 1, panel E-H was scanned by LSM700 to depict the localization of prestained hBMEC (red fluorescence), pericytes (green fluorescence) and astrocytes (blue fluroscence). The Z projections in X-Z directions are shown in top and right panel. The orthogonal planes of the X–Z and Y–Z projection are shown by the green and red lines respectively. It is advised to use ImageJ (https://imagej.net/ij/) for proper visualization of fluorescent signals. [file Image_2.tif]
